# New insights into the mechanisms underlying 5-fluorouracil-induced intestinal toxicity based on transcriptomic and metabolomic responses in human intestinal organoids

**DOI:** 10.1007/s00204-021-03092-2

**Published:** 2021-06-20

**Authors:** Daniela Rodrigues, Terezinha de Souza, Luke Coyle, Matteo Di Piazza, Bram Herpers, Sofia Ferreira, Mian Zhang, Johanna Vappiani, Daniel C. Sévin, Attila Gabor, Anthony Lynch, Seung-Wook Chung, Julio Saez-Rodriguez, Danyel G. J. Jennen, Jos C. S. Kleinjans, Theo M. de Kok

**Affiliations:** 1 Department of Toxicogenomics, GROW School for Oncology and Developmental Biology, Maastricht University, Maastricht, The Netherlands; 2 Departmnet of Nonclinical Drug Safety, Boehringer Ingelheim Pharmaceuticals Inc, Ridgefield, CT USA; 3 Present Address: F. Hoffmann-La Roche AG, Basel, Switzerland; 4 OcellO B.V., BioPartner Center, Leiden, the Netherlands; 5 Certara UK Limited, Simcyp Division, Sheffield, S1 2BJ UK; 6 GSK Functional Genomics/Cellzome, 69117 Heidelberg, Germany; 7 Faculty of Medicine, Heidelberg University Hospital, Institute for Computational Biomedicine, Heidelberg, Germany; 8 GSK Non-Clinical Safety, Ware, SG12 0DP UK; 9 Faculty of Medicine, Joint Research Centre for Computational Biomedicine (JRC‐COMBINE), RWTH Aachen University, Aachen, Germany; 10 Molecular Medicine Partnership Unit, European Molecular Biology Laboratory, Heidelberg University, Heidelberg, Germany

**Keywords:** 5-FU toxicity, Human organoid models, Molecular mechanisms, Transcriptomics, Metabolomics

## Abstract

5-Fluorouracil (5-FU) is a widely used chemotherapeutical that induces acute toxicity in the small and large intestine of patients. Symptoms can be severe and lead to the interruption of cancer treatments. However, there is limited understanding of the molecular mechanisms underlying 5-FU-induced intestinal toxicity. In this study, well-established 3D organoid models of human colon and small intestine (SI) were used to characterize 5-FU transcriptomic and metabolomic responses. Clinically relevant 5-FU concentrations for in vitro testing in organoids were established using physiologically based pharmacokinetic simulation of dosing regimens recommended for cancer patients, resulting in exposures to 10, 100 and 1000 µM. After treatment, different measurements were performed: cell viability and apoptosis; image analysis of cell morphological changes; RNA sequencing; and metabolome analysis of supernatant from organoids cultures. Based on analysis of the differentially expressed genes, the most prominent molecular pathways affected by 5-FU included cell cycle, p53 signalling, mitochondrial ATP synthesis and apoptosis. Short time-series expression miner demonstrated tissue-specific mechanisms affected by 5-FU, namely biosynthesis and transport of small molecules, and mRNA translation for colon; cell signalling mediated by Rho GTPases and fork-head box transcription factors for SI. Metabolomic analysis showed that in addition to the effects on TCA cycle and oxidative stress in both organoids, tissue-specific metabolic alterations were also induced by 5-FU. Multi-omics integration identified transcription factor E2F1, a regulator of cell cycle and apoptosis, as the best key node across all samples. These results provide new insights into 5-FU toxicity mechanisms and underline the relevance of human organoid models in the safety assessment in drug development.

**Supplementary Information:**

The online version contains supplementary material available at 10.1007/s00204-021-03092-2.

## Introduction

5-Fluorouracil (5-FU) is a chemotherapeutic agent that belongs to the class of fluoropyrimidines, the fluorinated derivatives of pyrimidines, and to the group of antimetabolite drugs. Fluoropyrimidines were developed in the 1950s (Longley et al. 2003) and are still one of the most widely used class of anticancer drugs. Despite the severe adverse reactions (ADRs) that some patients experience (Chang et al. 2012), these compounds, and particularly 5-FU, are standard treatments for breast and colorectal cancers. Treatment with 5-FU often results in myelosuppression, dermatitis, cardiac toxicity (Chang et al. 2012; Gradishar and Vokes 1990), and in 40–80% of patients it may cause toxicity in the gastrointestinal tract, which particularly manifests as mucositis-related symptoms (Gibson and Bowen 2011; Sonis et al. 2004). Intestinal mucositis manifests as damage to the mucous membrane and loss of mucosal integrity (Gibson and Bowen 2011), predisposing patients to inflammation, infections, or even sepsis (Rubenstein et al. 2004). Furthermore, intestinal mucositis is usually accompanied by nausea, abdominal pain, vomiting and diarrhoea, which has a major impact on health and the quality of life of patients. These ADRs and other complications, namely dose-limiting diarrhoea, may lead to the interruption or alteration of the cancer therapy (Dore et al. 2017).

Although the mechanisms of 5-FU-induced toxicity are not yet fully understood, its pharmacological mechanism of action is known. 5-FU, being an analogue of uracil, is readily transported into the cells, where it is converted into several active metabolites that can interfere with the function of thymidylate synthase (TS), an enzyme responsible for nucleotide synthesis, as well as the synthesis and function of DNA and RNA by incorporation in these molecules (Lee et al. 2014; Longley et al. 2003). Activation of p-53 expression by 5-FU was also observed in cells of intestinal crypts, which in turn lead to both inhibition of proliferation and induction of apoptosis (Pritchard et al. 1998). 5-FU and its metabolites can also lead to the production of reactive oxygen species (ROS), with subsequent release of pro-inflammatory cytokines, and platelet-activating factors (PAF), causing inflammation of the intestinal tissue (Logan et al. 2007; Soares et al. 2011). Current diagnosis or predictive tests of the ADRs induced by 5-FU in the intestinal mucosa are still unreliable, invasive and challenging, as they rely on endoscopies, biopsies or even patients’ own reports.

Gastro-intestinal safety and toxicological assessment during development of drugs conventionally relies on experiments with animal models to investigate molecular mechanisms and biomarkers that are related to acute or chronic toxicity (Cook et al. 2014). Nevertheless, the ethical considerations to reduce the number of animal experiments and the poor translation between animal, mainly rodents, and human in vivo gut cells’ responses, limit the investigation on drug-induced intestinal toxicity (Lanas and Sopena 2009; Monticello et al. 2017) and emphasize the need for human relevant test systems. Standard 2D cell-based assays are widely used to perform toxicity studies, despite their poor reflection of human tissue responses. One of the most common examples is Caco-2 cells, enterocyte-like cells that are derived from human colorectal adenocarcinoma, whose differences in the expression of enzymes and transporters hampers the direct extrapolation to the in vivo situation (Sun et al. 2008). Therefore, a novel in vitro culture system has been established based on 3D culture of organoids (Sato et al. 2009) that may better reflect human in vivo responses. Organoids present similarities in proliferation, differentiation and functional behaviour with human in vivo cells and tissues (Barker et al. 2010; Grabinger et al. 2014; Huch et al. 2013; Sato et al. 2009), giving them a potential advantage over 2D in vitro models. Since human organoids may better represent the intestinal physiology as compared to 2D cultures, their application in translational research could be a good alternative to the current in vitro intestinal models. The use of intestinal organoids has increased exponentially in recent years and has shown potential for the investigation of intestinal development, homeostasis, and human disease development. However, these organoids have not yet been fully explored for the evaluation and screening of drug toxicity. Therefore, this study included a 3D culture of intestinal human organoids to better understand intestinal toxicity induced by 5-FU.

Human intestinal organoids used in the present work were derived from human biopsies of the colon and small intestine (SI), and acquired intestinal-like structures after being cultured with specific growth factors (Sato et al. 2011). Relevant exposure concentrations were established using physiologically based pharmacokinetic (PBPK) modelling and simulation of clinical dosing regimens for 5-FU (Ferreira et al. 2020; Hanke et al. 2018; Kuepfer et al. 2018; Tylutki et al. 2018). Intestinal organoids were exposed to 3 different concentrations during 24 h, 48 h and 72 h of treatment. Cytotoxicity parameters for cell viability, based on ATP quantification, and apoptosis, based on caspase 3/7 activation, were measured. Nuclear and actin cytoskeletal imaging analysis were performed to characterize cell viability and structural morphology. Furthermore, in-depth quantitative RNA-sequencing and metabolomic analysis were performed to evaluate global changes in gene expression and metabolome profiles, respectively. Integration of both transcriptomics and metabolomics was ultimately performed in order to have a more detailed insight on 5-FU effects, as well as to check if gene and metabolomic responses are closely related. This study aimed at evaluating whether human intestinal organoids are a suitable model to investigate the mechanism of action underlying 5-FU induced intestinal toxicity by identifying differentially expressed genes (DEGs) and the most affected biological pathways, which can ultimately be translated to functional endpoints and human responses.

## Materials and methods

### 3D in vitro culture of healthy intestinal organoids

Human healthy colon organoids were kindly provided by Boehringer Ingelheim Pharmaceuticals Inc. (Ridgefield, USA), whereas human healthy SI organoids were generated and cultured in the Boehringer Ingelheim laboratory (Ridgefield, USA). Colon and SI organoids were derived from a tissue biopsy of the respective regions, performed on different healthy male donors. Establishment of the 3D in vitro culture of the colon and small intestine tissue was adapted from the methods described by Sato et al*.* (Sato et al. 2011, 2009). Initiation of intestinal organoids culture from tissue samples was based on EDTA chelation to allow collection of crypts (Sato et al. 2011, 2009; VanDussen et al. 2015). Isolated crypts were seeded at approximately 500 crypts/clumps per 50 uL Matrigel/well in a 24-well plate, and 200µL of complete crypt medium composed of advanced Dulbecco’s modified eagle medium (DMEM)/F12 medium (Life Technologies), Wnt3a conditioned medium, 1 µg/mL recombinant Human R-Spondin-1 (Peprotech), 10 mM nicotinamide (Sigma-Aldrich), 1 × B27™ Supplement (50x) serum free (Invitrogen), 1,25 mM N-acetyl-l-cysteine (Sigma-Aldrich), 50 ng/ml recombinant Human HB-EGF (Peprotech), 0,5 µM A 83–01 (Tocris), 10 µM SB 202190 (p38i) (Sigma-Aldrich), 10 µM human [Leu^15^]-Gastrin I (Sigma-Aldrich), 1 × Primocin (Fisher Scientific), 0,1 µg/mL recombinant human Noggin (Peprotech) and 10 µM inhibitor Y-27632 (AbMole bioscience) until the plate showed high confluency and signs of cell differentiation, i.e. changes in the morphology of the organoids. Afterwards, culture of organoids was transferred to 96-well plates.

Intestinal organoids were passaged every 3–7 days, depending on the rate of growth and/or changes in their morphology (differentiation). Culture plates containing organoids were firstly put on ice to promote easier disruption of the Matrigel matrix (phenol-red free, Corning) and washed with cold basal culture medium composed of advanced DMEM/F12 medium, Glutamax 100x, HEPES buffer and foetal bovine serum (FBS), 10% (v/v) (Life Technologies). Organoids were collected into 15-ml conical tubes and centrifuged at 300×*g* for 5 min, at 4 °C. Afterwards, the pellet was re-suspended in Tryple Express 1x (Life Technologies) containing inhibitor Y-27632 (AbMole bioscience), followed by a quick vortex and 2 min incubation at 37 °C. Basal culture medium was then added to the tube and organoids are dissociated into single cells. Two more centrifugations at 800×*g* for 5 min, 4 °C, were performed to wash the pellet, which was re-suspended in ice-cold Matrigel. Drops of 10 µL of Matrigel containing organoids were seeded in pre-warmed culture plates and polymerized at 37 °C for 15–20 min. Pre-warmed complete crypt medium was added to each well and incubated at 37 °C, 5% CO_2_. The medium was refreshed every 2–3 days. Before exposure to the drug, both organoids were also grown in Human IntestiCult™ Organoid Growth Medium (Stemcell), prepared according to manufacturer’s instructions, to mainly promote organoid differentiation.

### Selection of 5-FU in vitro concentrations

The Simcyp® Human PBPK simulator (v18r2; Certara UK Ltd., Sheffield, UK) and the Simcyp Sim-Cancer population were used to develop and verify a model describing 5-FU PK in cancer patients following IV dosing. Physicochemical properties and experimentally derived plasma protein binding, clearance and distribution data were collected from the literature for the PBPK model development (Supplemental Table S1). The M-ADAM (Multi-layer gut wall) model, available within Simcyp’s advanced dissolution, absorption and metabolism (ADAM) model was used to predict the enterocyte drug concentration in addition to the systemic plasma levels. Two different 5-FU clinical dosing regimens recommended for adult patients with colorectal cancer were simulated: regimen A—500 mg/m^2^ by IV bolus on Days 1, 8, 15, 22, 29, and 36 in 8-week cycles and regimen B—400 mg/m^2^ by IV bolus on Day 1, followed by 2400 mg/m^2^ as a continuous IV infusion over 46 h every 2 weeks (Drugs.com 2018). Systemic plasma and enterocyte in vivo concentrations were simulated for both dosing regimens. The VIVD model (Fisher et al. 2019) implemented in Simcyp’s In Vitro Data Analysis Toolkit (SIVA v3.0, Certara UK Ltd., Sheffield, UK) was used to model the in vitro distribution of 5-FU in human gut epithelial cells and predict the nominal test concentrations to achieve intracellular concentrations equivalent to those simulated using PBPK. The input parameters for this model can be found in Supplemental Table S2.

### In vitro exposure to 5-FU

5-Fluorouracil (5-FU) was purchased from Sigma-Aldrich (Germany), with ≥ 99% purity. Intestinal organoids were seeded in 96-well plates in complete crypt medium for 2 days. Afterwards, complete crypt medium was replaced with Human IntestiCult Growth medium, in which organoids remained for 3 days to stimulate differentiation. Differentiated intestinal organoids were exposed to 100 µL Human IntestiCult Growth medium with 10 µM, 100 µM and 1000 µM 5-FU for 0 h, 24 h, 48 h and 72 h, in repeated doses, i.e. medium was changed every 24 h. The selected concentrations were within the range of therapeutic doses as described in Sect. 2.2. Time points were selected based on previous experiments to evaluate the development of toxicity and transcriptomic responses. Vehicle controls, consisting of organoids seeded in 100 µL IntestiCult medium with 0,33% DMSO, and untreated controls, consisting of organoids seeded in 100 µL IntestiCult medium only, were also included for all time points. All samples were performed in technical triplicates in 96-well plates. A blank reaction was added to the treatment layout, consisting of Matrigel without organoids in 100 µL IntestiCult medium. Empty wells were filled with 200 µL of Phosphate-buffered saline (PBS) to avoid edge effects. After exposure, samples were collected to perform toxicity assays and transcriptomic analyses.

### Cytotoxicity assays: ATP measurement and 3/7 caspase activity

Measurement of the toxicity profile was performed using viability (ATP measurement) and apoptosis (3/7 caspase activity) endpoints kits 3D Celltiter-Glo and Caspase-Glo 3/7 (Promega), respectively. After each time point of exposure, medium was removed from the plates and replaced by 100 µL of each kit reagent to the appropriate wells, followed by homogenization of the Matrigel. The plates were placed in a Scilogex MX-M 96 well plate shaker for 1 h (incubation time), at room temperature. Afterwards, samples were transferred to white opaque 96-well plates (Corning) and luminescence was measured in GloMax® 96 Microplate Luminometer (Promega). Luminescence values corresponding to the levels of either ATP or 3/7 caspase activation, were transferred to Excel and corrected for the blank reaction to eliminate possible interferences of the Matrigel matrix in both curves.

### Image analysis

Image-based morphological analysis of the organoids was performed in the 3D image analysis solution Ominer® (OcellO B.V.). For this, organoids were grown in Matrigel for a total of 6 days during which they were exposed to 5-FU for 1, 2 or 3 days. After each time point of exposure, fixation and staining were performed to visualize the nuclei and actin cytoskeleton, by applying a solution containing Hoechst 33,258 (Sigma-Aldrich; final concentration: 0.4 µg/mL) and rhodamine–phalloidin (Sigma-Aldrich; final concentration: 0.1 µM) (Di et al. 2014). Untreated controls and vehicle controls were also included. Images were captured as z-stacks using a 4 × objective of ImageXpress Micro XLS (Molecular Devices) wide field microscope.

### RNA isolation from intestinal organoids

At each time point, medium was removed from the plates and 200 µL of QIazol Lysis reagent (Qiagen, The Netherlands) was added into the wells, followed by dissociation of the Matrigel matrix and transfer of each sample to the respective tubes. This process was repeated several times, until all organoids were collected, and ensuring a total volume of QIazol Lysis reagent of 700 µL. Complete homogenization of organoids in the lysis reagent was reached by vigorous pipetting and vortex. RNA isolation was performed using the miRNeasy Mini Kit (Qiagen, The Netherlands), following manufacturer’s protocol for Animal Cells including a DNase treatment. Total RNA yield and quality (assessed by 260/230 and the 260/280 ratios) were measured on a Nanodrop® ND-1000 spectrophotometer (Thermo Fisher, The Netherlands). The integrity of total RNA was confirmed using RNA Nanochips on a 2100 Bioanalyzer (Agilent Technologies, The Netherlands). All samples with integrity number (RIN) > 6 and total amount of RNA ≥ 200 ng were approved for RNA sequencing, which was the case for all the samples generated. Samples presented on average a RIN of 8.2 ± 0.5 and total RNA of 1134.8 ng and 1746.7 ng for colon and SI organoids, respectively.

### Library-preparation and mRNA sequencing

Samples containing purified RNA were prepared for sequencing using Lexogen SENSE mRNA library preparation kit (Lexogen, The Netherlands). After library preparation, the samples were sequenced on the HiSeq2500 (100 bp paired-end). A pool of all 5-FU samples and controls was sequenced on all 8 lanes of a flow cell. Untreated control, vehicle control and 5-FU samples were sequenced with an average of 10–15 million raw reads. One replicate from the colon organoids samples (10 µM 5-FU, 24 h) was excluded from the analyses due to low read counts (< 1,000 read counts).

### Pre-processing and data analysis

For all samples, due to the fact that the sequencing reads still contained Lexogen adapter sequences, the first 12 bases of the 5’end of all reads were removed using Trimmomatic version 0.33 (Bolger et al. 2014). Before and after trimming, the quality of the sequencing data was confirmed using FastQC version 0.11.3 (Andrews 2014) and only samples with satisfactory parameters were kept for downstream analysis. Following trimming, reads were aligned to the primary assembly of the human genome (Ensembl build v. 93 GRCh38) using Bowtie 1.1.1 and quantified with RSEM 1.3.1. The profile and behaviour of the samples were assessed according to the amount of (mapped) reads, hierarchical clustering, principal component analysis (PCA), and sample dispersion. Following quantification of the read counts, normalization was performed on the expected read counts from all samples and the contrast function from the R package DESeq 2 (v. 1.14.1) (Love et al. 2014) was used to extract DEGs for each time point and concentration so that comparison between all conditions of the experiment is possible. For each specific time point, the following comparisons were performed: (a) Untreated control vs Vehicle control; (b) 10 µM 5-FU vs Vehicle control; (c) 100 µM 5-FU vs Vehicle control; (d) 1000 µM 5-FU vs Vehicle control. Bonferroni correction (Aickin and Gensler 1996) was applied to the genes obtained, after which genes with *q* < 0.05 were considered as DEGs.

### Pathway analysis

The lists of DEGs obtained for each time point and concentration were used as input for pathway over-representation analysis (ORA) using CPDB release 34 (Kamburov et al. 2013), considering a cut-off of 0.01, *p* value ≤ 0.05 and *q* value < 0.05. The Reactome database version 67 (Fabregat et al. 2018) and Kyoto Encyclopedia of Genes and Genomes (KEGG) (Du et al. 2014) were selected as preferred databases for pathway analysis and interpretation of biological processes. The most relevant DEGs involved in cell cycle and apoptosis were visualized using PathVisio 3.3.0 (Kutmon et al. 2015) to aid in the interpretation. Gene plots of the most relevant DEGs were also generated using Excel. Moreover, the STEM tool (Ernst and Bar-Joseph 2006) was used in order to classify the DEGs according to their differential expression degrees during the exposure to 5-FU, considering a *p* value ≤ 0.05. For genes that were not differentially expressed in all the concentrations and durations of treatment in both colon and SI organoids, the missing expression value was assigned as zero. Clusters of time-dependent DEGs were ranked according to their significance (*p* value), after which only the top 3 significant gene clusters were chosen for further analysis. The list of genes obtained from the most significant clusters in the STEM tool were further analysed by performing a List Enrichment Network using the NetworkAnalyst 3.0 (Zhou et al. 2019).

### Metabolomic analysis of human organoids supernatant

For each condition, 100 µL of media were collected and stored at − 80 °C until analysis. Prior to analysis samples were thawed at room temperature for 30 min and diluted tenfold in LC/MS-grade water. Non-targeted flow injection-mass spectrometry spectroscopy analysis of polar metabolites was performed as described in (Fuhrer et al. 2011). Ten µL of each sample were injected in a randomized sequence using an Ultimate-3000 liquid chromatography systems (Thermo Scientific) and analysed in negative ion mode on a Q-Exactive mass spectrometer (Thermo Scientific) using a mobile phase consisting of 60% isopropanol, 40% water, 1 mM NH_4_F, 10 nM taurocholic acid and 20 nM homotaurine. The mass spectrometer was calibrated according to manufacturer protocols and operated in profile mode in a scan range of 50–1000 *m*/*z*. Resolution was set to 70,000 at 200 *m*/*z* with automatic gain control target of 3E6 ions, 3.0 kV spray voltage, 120 ms maximum injection time, and 60 s acquisition time. Quality control samples were injected every 20 runs to ensure proper instrument performance. Peak detection and global alignment of all scans was performed using a custom metabolomics data processing pipeline. To tentatively annotated metabolites, detected ion *m*/*z* values and isotope distributions were matched against the Human Metabolome Database assuming [M-H] and [M-2H] species and at most two 13C/12C exchanges, with the method-inherent limitation of being unable to distinguish between isomers. Statistical analysis of the data was performed using R. Statistical significance between compared sample groups was assessed using unpaired t-tests, and resulting *p* values were adjusted for multiple hypothesis testing using Bonferroni’s method.

### Multi-omics integration

#### Transcription factor activity estimation

A footprint based approach (Dugourd and Saez-Rodriguez 2019) was used to estimate transcription factor activity. First, the log2-fold change RNA-sequencing data was processed and genes that were not expressed in the basal condition (determined from the raw counts) were removed, which would have led to an arbitrarily large fold change. This resulted in 16,552 and 15,045 genes in SI and colon samples, respectively. To determine the transcription factor activity in each sample, the VIPER algorithm (Alvarez et al. 2016) was used with the DOROTHEA regulons (Dugourd and Saez-Rodriguez 2019; Garcia-Alonso et al. 2019) as implemented in the `dorothea` R package (version 1.2.0, available from Bioconductor).

In summary, the algorithm ranked the genes by their fold changes, then derived an enrichment score for each transcription factor based on the rank of their targeted genes. In this scoring method, the mode of action (i.e. up- or downregulation of targets) was also considered. Afterwards, this score was compared to a null-distribution of scores that were derived from randomized gene sets with the same size as the size of the regulon (Z-transformation). All the samples were provided to construct the null-distribution of enrichment scores. This way the normalized enrichment score of the transcription factors could be compared across samples.

#### Multi-omics integration

COSMOS (Dugourd et al. 2020) was also used to integrate the transcriptomics and metabolomics data in a unified interaction network. COSMOS builds on a signed, directed prior knowledge network (PKN) that aggregates three main interaction resources: (1) Omnipath (Turei et al. 2016) for protein–protein interactions, STITCH (Szklarczyk et al. 2016) for allosteric regulations, and (3) RECON3D for human metabolic interactions (Brunk et al. 2018).

First, a specific prior knowledge network was created for the colon and SI samples separately. For that purpose, the expressed genes in each sample were identified separately using edgeR (Robinson et al. 2010), followed by removal of the interactions that contain proteins which were not expressed in any conditions of the organoid samples. Afterwards, the transcription factors and the measured metabolites were mapped to the prior knowledge network. In total, 391 of the 2538 measured metabolites were found in the PKN, from which 319 were significantly changing in abundance in at least one sample. Afterwards, the optimization step of COSMOS was run ten times for each sample to explore a family of networks. In this optimization step, the algorithm finds a subset of the PKN that contains the maximum number of inputs (transcription factors) and outputs (measured metabolites) and it connects these nodes using the minimum number of edges (further details in (Liu et al. 2019)). Each optimization run reported between one and one hundred equivalent solutions. The final objective function values of the independent optimization runs were similar (less than 5% difference), therefore the quality of the solutions was also similar. These solutions were aggregated by taking the union of the interactions reported in each solution. Then the essentiality of each edge was computed by the relative number of times it appeared across the solutions, i.e. the essentiality of 1 means that the edge appeared in all the solutions.

## Results

### Establishment of healthy human intestinal organoids

**Fig. 1:**
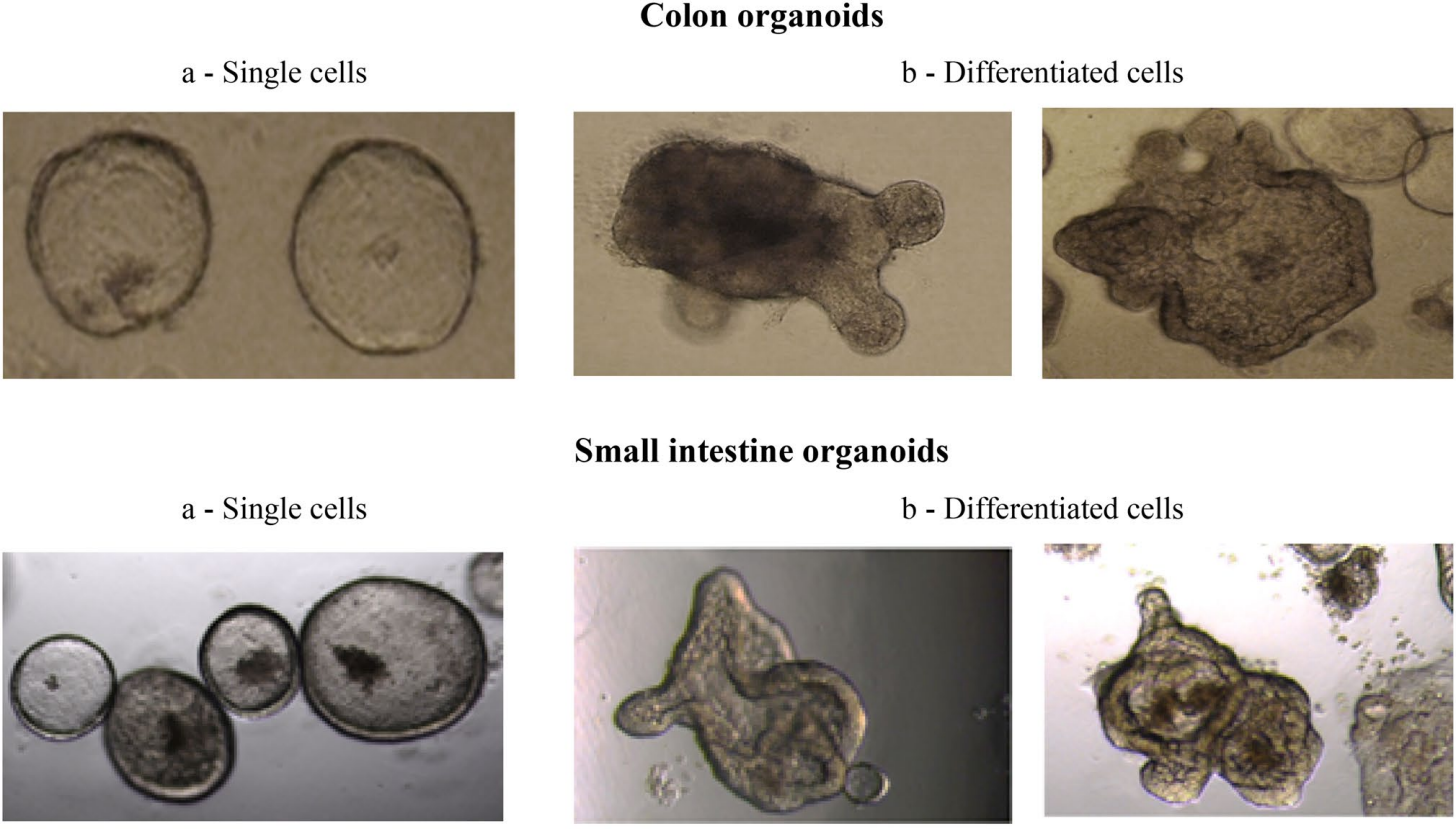
Images of colon and small intestine organoids obtained two distinct phases of development: a single, undifferentiated organoids with no signs of budding/crypt-like formation; b differentiated organoids with evident signs of budding/crypt-like formation. Total magnification of 200×

Organoids derived from biopsies of the SI and colon of healthy human donors were successfully generated in a 3D culture matrix. Both types of organoids showed fast growth and proliferation at the beginning of culture until 8–10 passages, after which organoid growth rate became stabilized. When the culture was started, organoids appeared as single undifferentiated cells, with a round shape. After approximately 3–4 days, organoids started to differentiate and form buds, which resembled intestine-like features (Fig. 1).

### Selection of 5-FU in vitro concentrations

**Fig. 2:**
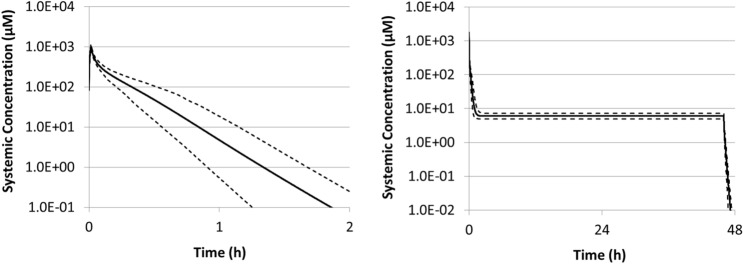
**a** Simulated (black solid line) systemic plasma concentration following a 500 mg/m^2^ IV bolus. **b** Simulated systemic plasma concentration profile (black solid line) for an IV bolus of 400 mg/m^2^ followed by 2400 mg/m^2^ IV infusion over 46 h. The dashed lines refer to the 5th and 95th percentiles of the virtual population simulated (10 trials with 10 cancer patients each, 50% female)

The performance of the 5-FU human PBPK model was validated using clinical observations (Supplemental Figure S1). Figure 2a shows the pharmacokinetics (PK) profile predicted for systemic plasma following dosing regimen A (500 mg/m^2^ intravenous (IV) bolus weekly). Since there was no pronounced systemic accumulation of 5-FU for regimen A (plasma *t*_1/2_ = 8–20 min) (Administration 1999), drug disposition was simulated for 24 h following the IV bolus.

Due to the short plasma half-life, 5-FU is increasingly being administered as an infusion to improve clinical efficacy (Morawska et al. 2018). The PK profile predicted for systemic plasma following the infusion regimen (regimen B—400 mg/m^2^ IV bolus for 24 h, plus 2400 mg/m^2^ IV infusion over 46 h every 2 weeks) is shown in Fig. 2b. The time profiles predicted for the intracellular enterocyte concentration in jejunum and colon have a similar shape to the ones obtained for systemic plasma (data not shown).

Table 1 shows the maximum drug concentration (*C*_max_) predicted for both dosing regimens and the steady-state concentration (*C*_ss_) achieved during infusion. While the *C*_max_ represents a more conservative ‘worst-case’ scenario considered in safety assessment, the *C*_ss_ provides complementary information on the concentrations achieved following repeated or continued drug exposure. The predicted 5-FU *C*_max_ is in the order of 10^3^ µM in plasma and 10^2^ µM in jejunum and colon enterocytes for both dosing regimens, while the 5-FU *C*_ss_ is 2–3 orders of magnitude lower. These predictions are in line with the colon concentrations measured by Peters et al. (1993), in the range of 0.04–22 µM, in the first hours following an IV bolus. However, prolonged tissue retention was observed in the Peters’ study. Literature data supporting 5-FU tissue accumulation is limited (Inomata et al. 1998; van Groeningen et al. 1988) and lacks a clear mechanistic explanation. For this reason, the PBPK model considers a relatively rapid exchange between plasma and tissue.

**Table 1:** Maximum (C_max_) and steady-state (C_SS_) concentrations simulated for systemic plasma, jejunum enterocytes and colon enterocytes

Dosing regimen	Plasma *C*_max_ (µM)	Plasma *C*_SS_ (µM)	Jejunum *C*_max_ (µM)	Jejunum *C*_SS_ (µM)	Colon *C*_max_ (µM)	Colon *C*_SS_ (µM)
(A) Bolus	1023		360		524	
(B) Infusion	1462	6.0	290	3.1	434	4.6

The values presented are for the mean profile of the population (10 trials with 10 subjects each)

The virtual in vitro intracellular distribution (VIVD) (Fisher et al. 2019) model was used to predict 5-FU distribution in vitro. Based on the physicochemical properties of the drug and assay conditions, this steady-state model predicts a ratio of 0.4 between the intracellular concentration and the nominal test concentration used in the assay. Therefore, nominal concentrations of 10, 100 and 1000 µM results in intracellular concentrations of 4, 40 and 400 µM, respectively. Following from this, while the lowest nominal concentration (10 µM) better replicates intracellularly the steady-state concentrations (*C*_ss_) achieved in vivo during 5-FU infusion, the highest concentration (1000 µM) better replicates the peak concentrations achieved in the gut immediately after an IV bolus (*C*_max_). Therefore, by combining PBPK modelling and VIVD, an in vitro testing strategy covering both predicted *C*_max_ and *C*_ss_ was designed.

### Cytotoxicity and functional assessment of organoids viability and apoptosis after exposure to 5-FU

**Fig. 3:**
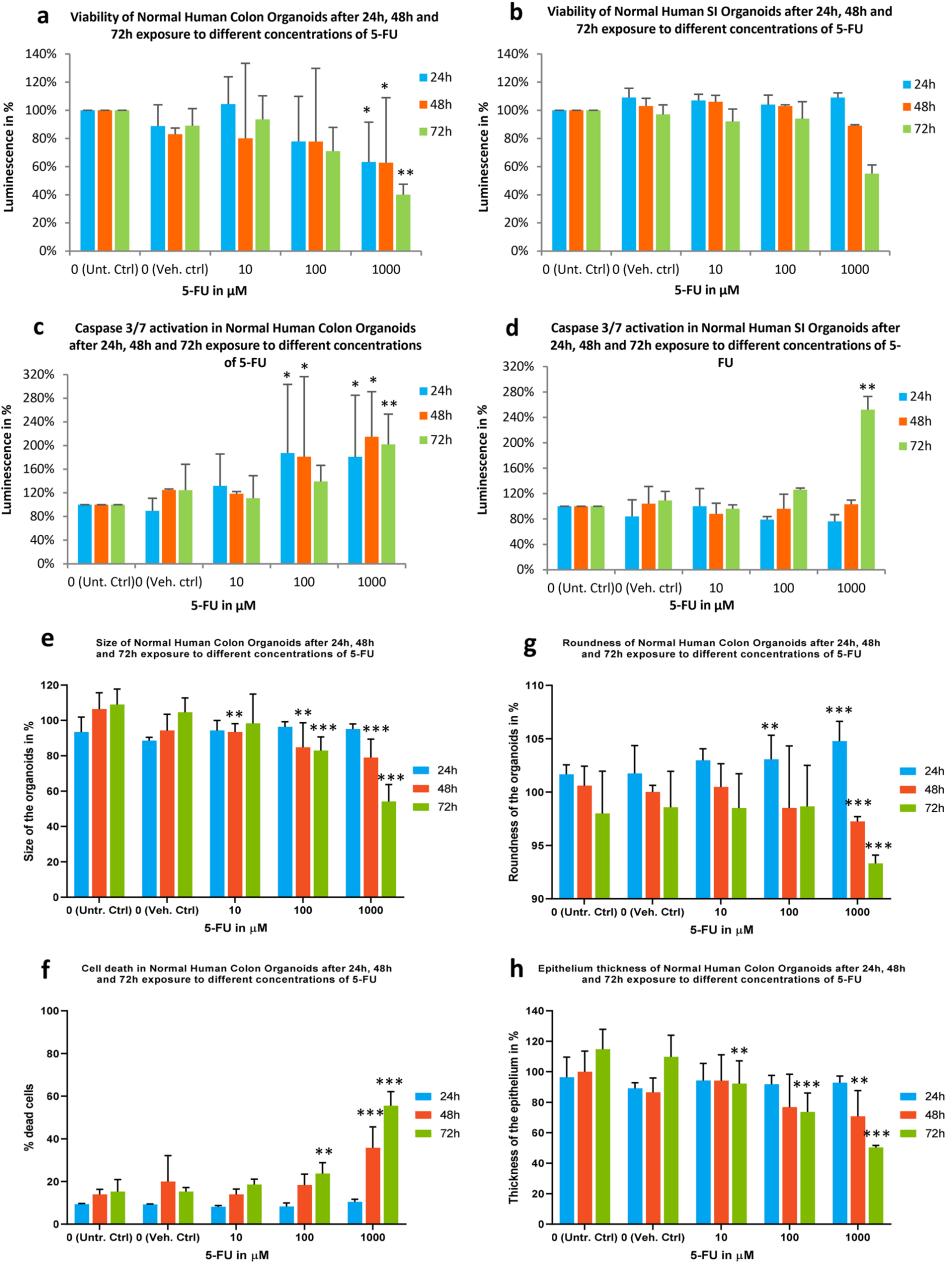

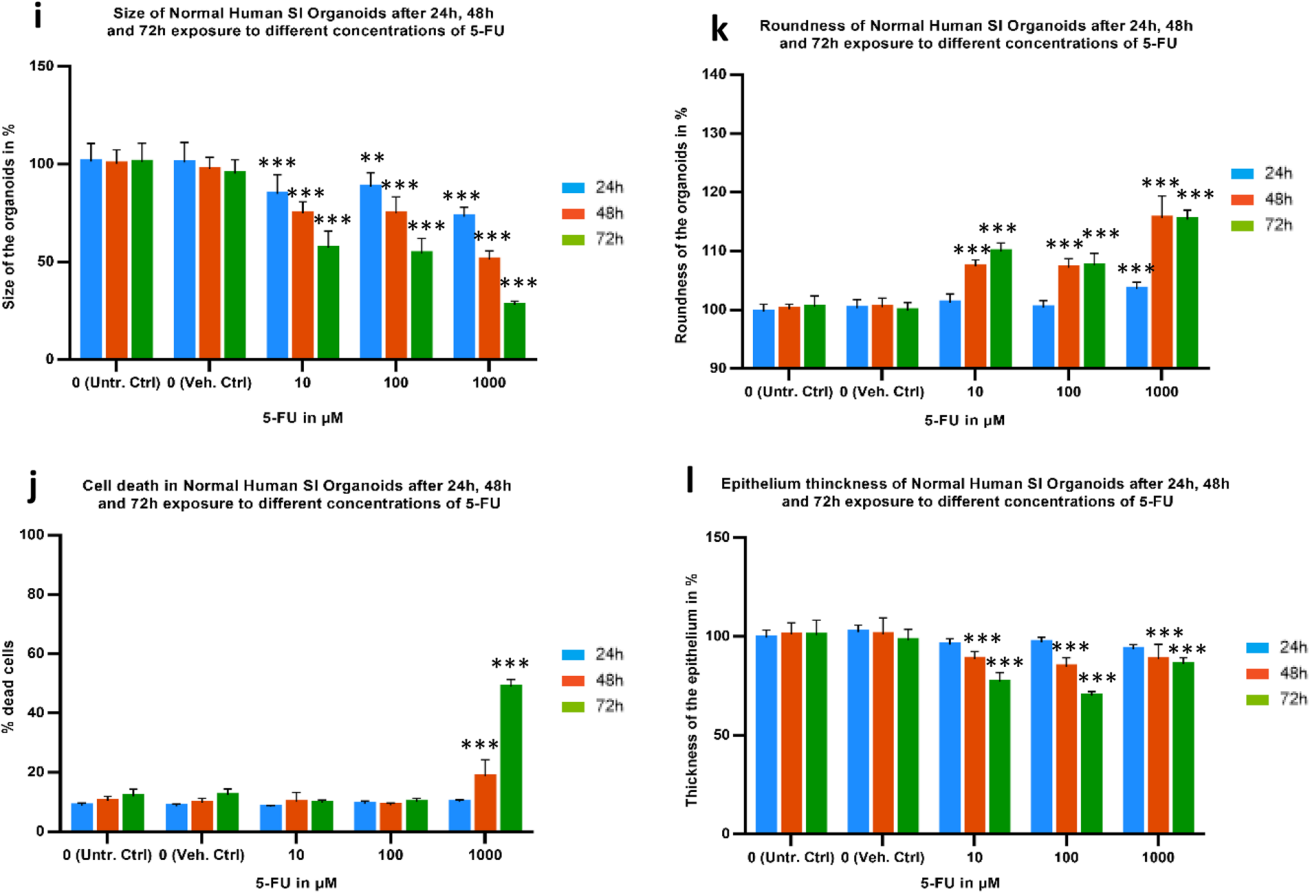
**a**–**d** Evaluation of viability determined by ATP levels and caspase 3/7 activation of healthy colon (**a** and **b**, respectively) and SI (**c** and **d**, respectively) organoids when exposed to 10, 100 and 1000 µM 5-FU for 24 h in blue, 48 h in orange and 72 h in green, compared with Untreated controls. Values are in % of luminescence. For each time point the average of Unt. Ctrl was set to 100%. **e** and **f** Morphological analysis of 5-FU-treated colon (**e**–**h**) and SI (**f**–**l**) organoids. 24 h 5-FU data on SI organoids is not available. SD was calculated for each condition. Legend: *Ctrl* control; *SD* standard deviation; *SI* small intestine; *Unt* untreated; *Veh* vehicle. *p value of 0.0011; **p value of 0.0009; ***p value < 0.0001 (color figure online)

As expected, ATP levels of 5-FU treated organoids decrease with concentration and time (Fig. 3a and b). For the low concentration (10 µM), there is no statistically significant difference between the time points and between treatments and the respective controls in both colon and SI organoid cultures. For the middle concentration (100 µM), there was a 20–30% decrease on ATP levels in colon organoids at all time points (*p* value of 0.023), but this was not observed in SI organoids. At the high concentration (1000 µM) there were further decreases in ATP levels at all time points in colon organoids, and from 48 h and above, in SI organoids. At 72 h, ATP levels had decreased by 60% in colon organoids (*p* value of 0.0009) and 45% in SI organoids (*p* value of 0.036).

For caspase 3/7 activation, which reflects apoptosis a positive trend was seen in colon organoids regarding both concentration and duration of treatment, particularly at the mid- and high concentrations (Fig. 3c). Indeed caspase 3/7 activation was twofold greater than untreated control levels in the high concentration group (*p* value of 0.0009). In contrast, caspase activation was not statistically significantly increased in SI organoids cultures below the high concentration or at any time point before 72 h (Fig. 3d, *p* value of 0.0009). These changes in cell viability and caspase 3/7 activation were accompanied by changes to the size and shape of both organoids, which became smaller, their roundness decreased, cell death increased and the epithelial cell layer became thinner over time for each concentration of 5-FU used (colon, Fig. 3e–h; SI, i–l).

### Analysis of in vitro transcriptomic responses induced by 5-FU

Gene expression data from 5-FU exposed organoids was used to identify biological pathways involved in 5-FU-induced intestinal toxicity. First, alignment of the samples against the whole human genome was performed, ranging between 70 to 89%, thus further analysis could be performed. From those analysis, it was observed that the number of DEGs consistently increased with treatment time and concentration of 5-FU (Supplemental Figure S2), for both colon and SI. Moreover, the number of DEGs was significantly higher in the colon organoids as compared to the SI at all time points and concentrations.

**Fig. 4:**
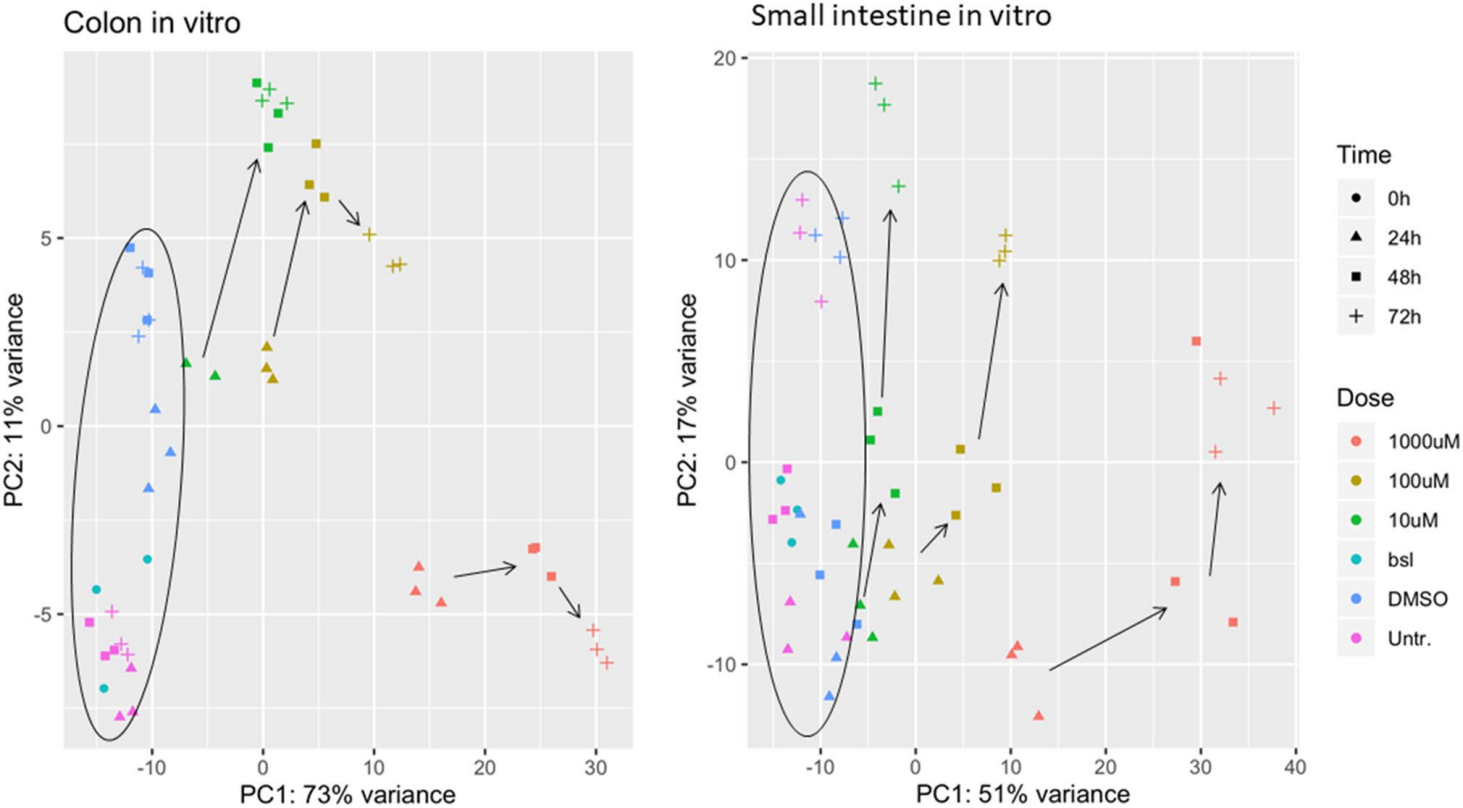
PCA scatter plot based on the mRNA transcriptomic analysis of samples collected from colon organoids (left) and SI organoids (right). Direction of the arrows indicates the evolution in time of the samples (24 h → 48 h → 72 h). The cluster on the left comprises non-treated samples (baseline at 0 h, untreated and vehicle controls at all time points). Legend: Baseline (0 h) is in light blue; untreated controls are in pink; Vehicle controls are in blue; 10 µM 5-FU is in green; 100 µM 5-FU is in yellow; and 1000 µM 5-FU is in orange (color figure online)

Furthermore, PCA scatter plots were created to explore differences between treated and untreated organoids based on the gene expression profiles affected by 5-FU concentration and treatment duration (Fig. 4). There was a clear separation between controls and treated samples and between 5-FU concentrations in both colon (Fig. 4 on the left) and SI organoids (Fig. 4 on the right). Measurements at baseline (0 h) and in controls (untreated or DMSO) from colon samples at all time points cluster closely together, whereas from SI samples there seems to be a higher influence of time. Duration is also influencing the separation of treated samples, except for the 10 µM colon samples at 48 h and 72 h, which are clustering together. Moreover, from the PCAs, we can observe that time of exposure is affecting the organoids more than the concentration as the variance is lower for the latter.

**Fig. 5:**
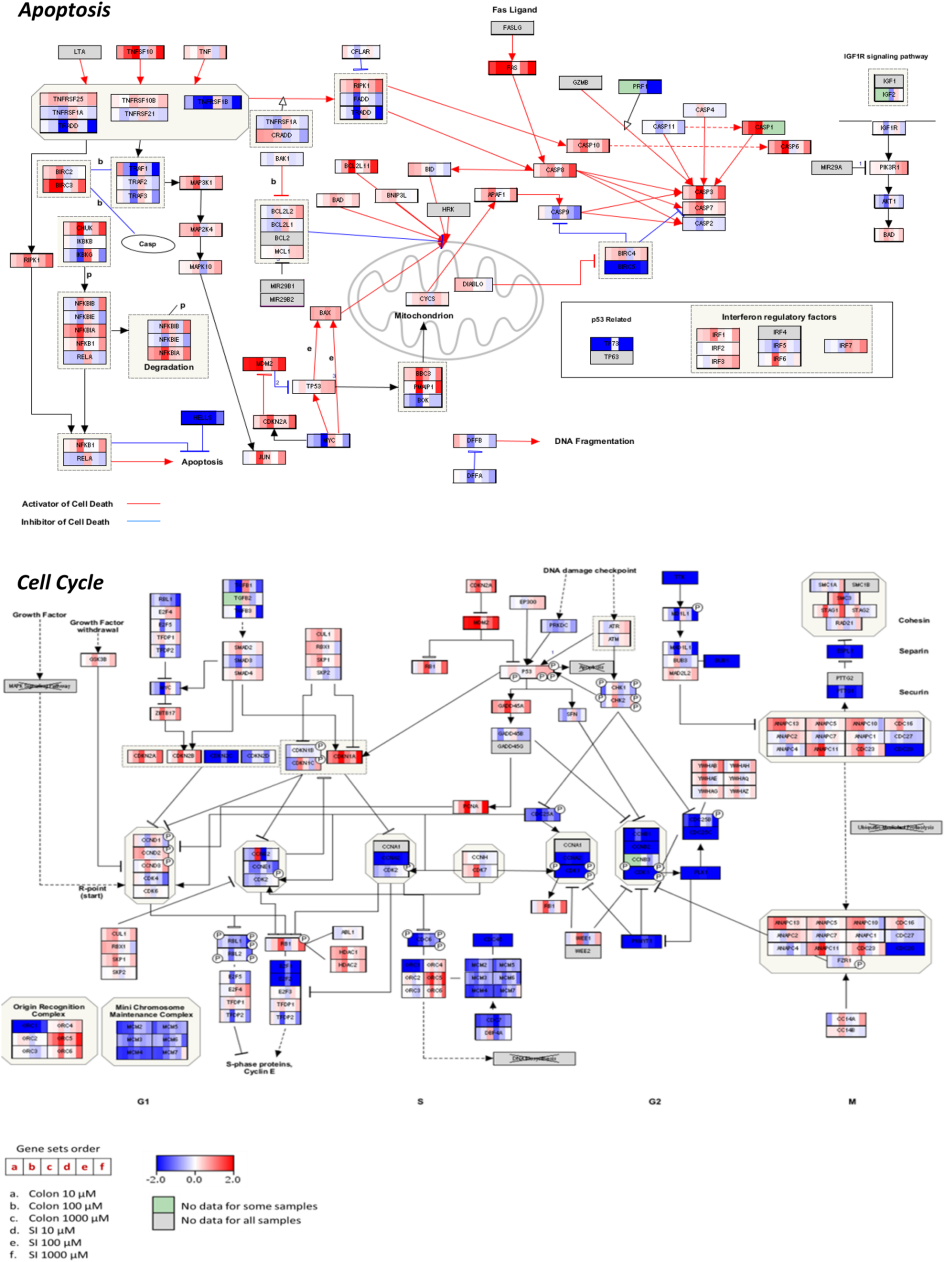
Pathway representation of apoptosis and cell cycle created with PathVisio based on transcriptomic data obtained from colon and SI organoids after 72 h exposure to different concentrations of 5-FU (10, 100 and 1000 µM). Blue colour represents downregulation and red colour represents upregulation. Colour gradient is based on the DEGs log2FC and indicates the strength of modulation

Subsequently, pathway overrepresentation analysis (ORA) was performed with Consensus Path Database (CPDB) (Kamburov et al. 2011) in order to obtain an overview of the altered pathways for treated samples compared to vehicle controls (DMSO) as there was no significant difference between the vehicle controls and untreated controls. Overall, 483 pathways were found altered in colon and 306 in SI organoids after exposure to 5-FU, of which the most significantly overrepresented pathways were identified using the *q* values and the number of DEGs involved. The pathways that were identified as more relevant over time and concentration of 5-FU in both organoids, considering lower *q* values and number of DEGs affected, are shown in Table 2; these include cell cycle, DNA synthesis, replication and damage response, respiratory electron transport and ATP synthesis, p53 signalling pathway, apoptosis and metabolism. From the number of DEGs affected by 5-FU (Table 2), it is apparent that, overall, the responses from the colon organoids are stronger than the SI ones. The visualization of alterations in the expression levels of transcripts associated with cell cycle and apoptosis DEGs was performed using PathVisio for colon and SI organoids exposed to 5-FU at 72 h (Fig. 5). These pathways were selected because, apart from being relevant for both colon and SI organoids response to 5-FU, they can be compared to the cytotoxicity and functional evaluations.

**Table 2:** Overview of the most relevant pathways and number of DEGs involved

Name of the pathway	Pathway source	Time of exposure (h)	5-FU concentration (µM)	*q* value		Total number of DEGs	
				Colon	SI	Colon	SI
Cell cycle	Reactome	24	10	3.49E-03	5.45E-12	9	14
			100	3.08E-14	2.46E-59	64	67
			1000	3.67E-03	4.87E-27	130	104
		48	10	2.77E-19	3.37E-46	75	57
			100	1.71E-17	5.72E-52	96	78
			1000	6.70E-06	4.97E-12	203	144
		72	10	1.44E-29	2.11E-40	94	49
			100	1.64E-09	5.60E-31	142	57
			1000	1.39E-10	6.70E-05	257	125
DNA replication	Reactome	24	10	NA	4.30E-06	NA	5
			100	1.75E-05	1.25E-19	10	18
			1000	2.08E-02	4.30E-08	25	21
		48	10	6.08E-05	2.27E-04	14	6
			100	8.36E-05	4.27E-08	18	12
			1000	1.87E-03	NS	37	11
		72	10	4.33E-09	1.88E-04	20	6
			100	3.41E-05	5.92E-03	30	6
			1000	2.16E-06	NA	49	NA
DNA synthesis	Reactome	24	10	NA	3.63E-06	NA	5
			100	5.99E-05	1.06E-18	13	17
			1000	9.48E-03	7.71E-08	25	20
		48	10	2.95E-05	1.33E-03	14	5
			100	3.28E-05	2.21E-07	18	11
			1000	4.84E-04	NA	37	NA
		72	10	9.27E-09	1.39E-04	19	6
			100	2.61E-05	4.49E-03	29	8
			1000	1.57E-06	NA	47	NA
DNA damage response	KEGG	24	10	1.46E-04	NA	5	NA
			100	6.06E-04	1.57E-10	11	11
			1000	4.10E-02	6.57E-07	21	18
		48	10	2.49E-04	7.04E-07	12	8
			100	5.53E-03	9.38E-06	13	9
			1000	2.15E-02	1.57E-02	29	20
		72	10	5.95E-07	9.42E-05	16	6
			100	1.63E-02	7.43E-06	20	9
			1000	1.15E-04	2.27E-02	40	20
p53 signalling pathway	KEGG	24	10	1.11E-07	8.30E-03	8	2
			100	2.46E-05	3.42E-09	13	10
			1000	7.21E-03	6.57E-07	24	18
		48	10	1.14E-05	7.04E-07	8	8
			100	5.80E-04	1.01E-06	15	10
			1000	3.63E-02	4.61E-04	28	24
		72	10	3.73E-06	9.42E-05	15	6
			100	3.26E-02	6.17E-05	6	2
			1000	2.62E-04	2.27E-02	11	20
Metabolism	Reactome	24	10	NA	NA	NA	NA
			100	2.18E-02	NA	92	NA
			1000	7.89E-08	NA	435	NA
		48	10	3.49E-03	NA	103	NA
			100	1.02E-04	NA	169	NA
			1000	5.58E-13	2.21E-04	658	331
		72	10	2.22E-03	NA	113	NA
			100	8.66E-11	NA	392	NA
			1000	2.87E-19	1.13E-06	806	368
Apoptosis	Reactome	24	10	1.58E-02	NA	3	NA
			100	7.31E-02	3.25E-02	10	4
			1000	4.30E-03	4.11E-03	37	17
		48	10	5.42E-02	4.99E-02	12	4
			100	2.94E-02	3.17E-02	20	5
			1000	8.01E-04	NS	52	24
		72	10	NA	4.25E-02	NA	4
			100	3.93E-02	5.10E-02	32	6
			1000	9.22E-04	9.33E-03	58	32
Respiratory electron transport and ATP synthesis	Reactome	24	10	NA	NA	NA	NA
			100	NA	NA	NA	NA
			1000	NA	NA	NA	NA
		48	10	NA	NA	NA	NA
			100	NA	NA	NA	NA
			1000	8.69E-03	NA	49	NA
		72	10	NA	NA	NA	NA
			100	2.80E-02	NA	30	NA
			1000	1.52E-04	NA	63	NA

Pathways obtained after identification of the DEGs using ORA with CPDB and ranked according to their *q* values. Alterations in these pathways can be observed over time and concentration of 5-FU. The *q* value was obtained after correction of the *p* values for multiple testing using the false discovery rate method. *q* values and total number of genes were considered as not applicable (NA) if not shown after ORA analysis

*KEGG* Kyoto Encyclopaedia of Genes and Genomes; *NA* not applicable; *NS* not significant

#### Pathway analysis over time and concentration in colon organoids

For the low concentration at the initial time point (24 h), the two most prevalent pathways are p53-related, namely transcriptional regulation by p53 and p53 signalling pathways. The first one is significantly affected early on in colon organoids, and at later time points (48 h and 72 h) is superseded with pathways associated with cell cycle. At 48 h, p53 signalling pathway remains one of the most important pathways affected along with cell cycle, which stood out at this time point. As cell cycle becomes more affected across treatment conditions, p53 signalling pathway becomes less affected, based on the respective *q* values (Table 2). At 72 h, cell cycle becomes more affected (lowest q-value) along with synthesis of DNA. Consequently, DEGs involved in those pathways are more downregulated. On the other hand, for the intermediate concentration, at 24 h and 48 h, perturbed pathways include cell cycle, DNA synthesis and replication as the most prominent. Noticeably, at 72 h, apart from cell cycle, metabolism (i.e. of amino acids and proteins, nucleotides, folate, lipids and carbohydrates, mainly glucose and pyruvate) becomes a more affected pathway over time as well. Finally, at the high concentration, metabolism remains as the most affected pathway in all the time points, with a q-value higher at 72 h.

Summarizing, the pathway analysis identified the modulated pathways in response to the toxic effects of 5-FU in a time and dose-dependent manner. Whereas initially p53 and cell cycle related pathways prevail as the most affected ones, at the highest concentration and latest time point, metabolism becomes most significantly affected.

#### Pathway analysis over time and concentration in SI organoids

Looking at the gene expression profile and pathways that are affected by 5-FU in SI organoids, cell cycle seems to be the most affected at all time points and concentrations, with q-values that are lower than those obtained in colon organoids (Table 2). At 10 µM, cell cycle becomes more affected over time, particularly from 24 to 48 h. Hence, most cell cycle genes become more downregulated as the concentration of 5-FU increases from 100 to 1000 µM and over time (from 24 h until 72 h).

In addition to cell cycle, other pathways that appear to be more significantly modulated in SI organoids are transcriptional regulation by p53 and DNA related pathways (replication, damage response and synthesis). Finally, metabolism is significantly affected at later time points, after exposure to 1000 µM only. Moreover, lower concentrations of 5-FU at all time points didn’t affect metabolism as it was observed for colon organoids (Table 2). Hence, biological pathways are similarly affected in colon and SI organoids, being the only difference the metabolic pathways, which are more affected and at earlier time points in colon than in SI (Table 2).

Overall, in both organoids, cell cycle related genes became more downregulated as the time and concentration of 5-FU increases. The decrease of cell cycle was accompanied by activation and increase of apoptosis at later stages, particularly at higher concentrations and latest time point (72 h). After 72 h of exposure to 1000 µM 5-FU the number of dead cells was also higher as compared to the other treatment conditions. The gene expression alterations of the cell cycle and apoptosis pathways are visualized with PathVisio as shown in Fig. 5, based on transcriptomic data of all 5-FU concentrations at 72 h exposure of both types of organoids. The colour-code in Fig. 5 demonstrates a clear concentration-dependent up- and downregulation of modulated genes.

#### Concentration-dependent gene expression responses involved in ATP synthesis and apoptosis

**Fig. 6:**
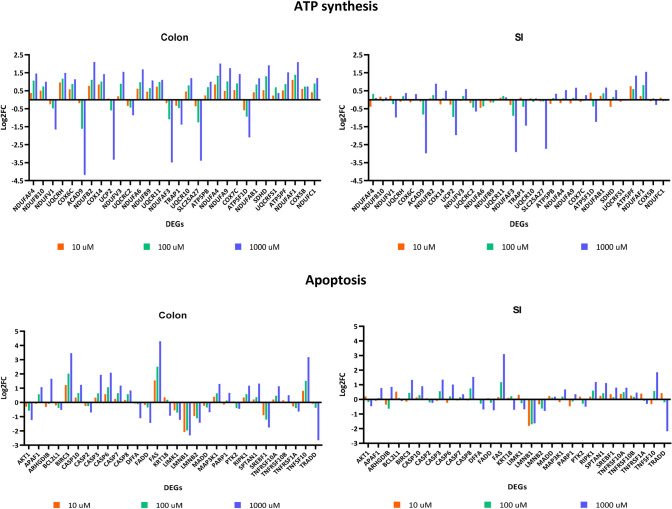
Gene plots summarizing the expression profiles of genes involved in ATP synthesis and apoptosis pathways after 72 h exposure to 5-FU of organoids derived from colon (on the left) and SI (on the right). Values for gene profiles are based on the log2FC. Plot colours correspond to the different concentrations of 5-FU: orange represents 10 µM; green represents 100 µM; and blue represents 1000 µM (color figure online)

In order to link the phenotypic data with the transcriptomic data, we correlated the results obtained in the viability, apoptosis and morphology assessment with the expression of genes involved in the pathways “Respiratory electron transport and ATP synthesis” and “Apoptosis”. For this analysis, the longest time of exposure (72 h) was selected since significant changes are more evident for both ATP synthesis and apoptosis. Gene plots were constructed using the log2 fold change of the DEGs involved in those pathways (Fig. 6). Regarding ATP synthesis pathway, gene plots demonstrate that the most affected genes in both organoids were *ACAD9, UCP2, NDUFAF3, SLC25A27* and *ATP5PB* (Fig. 6). In turn, most affected genes involved in apoptosis were pro-apoptotic, of which *FAS, TNFSF10* and *CASP3/6/7/8/10* stood out as the genes that were found more upregulated by 5-FU in both organoids (Fig. 6). Two anti-apoptotic genes were also found, namely *BIRC3, BCL2L1* and *AKT1,* being the first one upregulated and the latter ones down-regulated. Despite up-regulation of *BIRC3*, up-regulation of pro-apoptotic genes had a greater impact on cell’s fate.

Considering the cytotoxicity and morphology data, it was observed previously that colon organoids are more sensitive to 5-FU effects than SI organoids. The transcriptomic data confirm these observations by looking at the q-values of each pathway (Table 2) and the respective gene plots (Fig. 6). ATP levels and transcriptomic data show a clear concentration-dependent effect, particularly in the colon organoids. Likewise, caspase 3/7 activation, cell death data and upregulation of pro-apoptotic genes clearly show that cell death mechanisms are activated in response to the concentration-effect in both organs, but again more strongly in the colon.

Moreover, the results represented in Fig. 5 in combination with the gene plots (Fig. 6) confirm the concentration-dependent trends in the gene responses that were mentioned earlier. In general, apoptosis is becoming more activated due to the upregulation of most pro-apoptotic DEGs involved in both extrinsic pathway with caspases activation and intrinsic pathway mediated by mitochondria via upregulation of *BAX* gene. Furthermore, variance in the expression of apoptotic genes and their upregulation as the concentration of 5-FU increases is stronger in the colon that in the SI organoids. This is consistent with the results obtained in the caspase 3/7 activation, imaging assessment (Fig. 3) and the apoptosis gene plots (Fig. 6), confirming the stronger effect of 5-FU in activation of apoptosis in the colon organoids.

### Time-dependent gene clustering

**Fig. 7:**
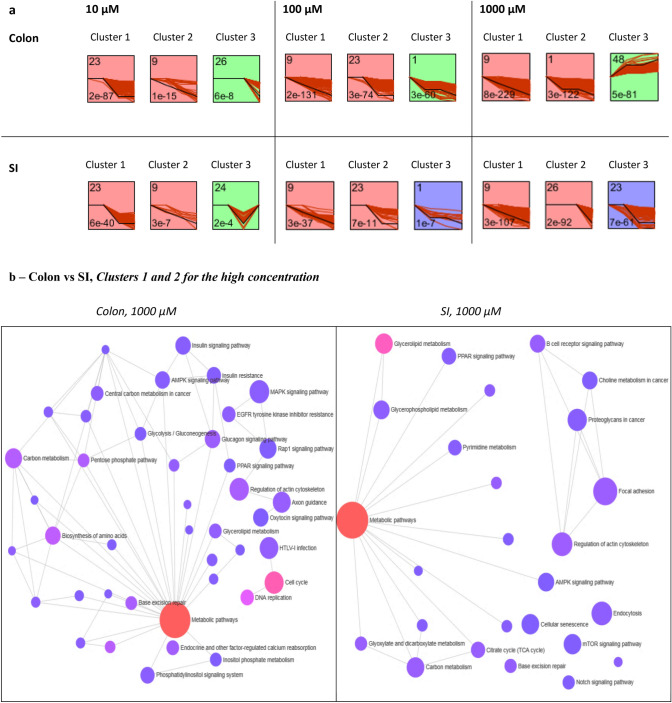
**a** Correlated expression clusters discovered using the short time-series expression miner (STEM) algorithm for time-series profile matching. Profiles were ordered based on *p* value significance of number of genes versus expected. The top 3 clusters were selected for each concentration of 5-FU in both organs; Legend: for each profile box, axis *x* correspond to time points and axis *y* to the gene expression change based on log2FC; number on the top left of a profile box is the profile ID number; number on the bottom left is the profile enrichment *p* value; background colour mean profiles have a statistically significant number of genes assigned and same colour represents profiles grouped into a single cluster; red lines represent the expression changes of the profile genes over time; **b** Functional annotation of DEGs of time-series clusters 1 and 2 for the highest concentration of 5-FU (1000 µM) in both colon and SI by performing list enrichment network in the NetworkAnalyst 3.0, based on KEGG pathways. Most relevant pathways can be observed for each organoid type (color figure online)

To further investigate the dynamics in gene expression profiles, a time-series correlation analysis was performed on the DEGs of all time points (0, 24, 48 and 72 h) for each condition (concentration of 5-FU), using the Short Time Series Expression Miner (STEM) tool (Ernst and Bar-Joseph 2006). Several clusters of time-dependent DEGs were observed and some conditions presented more than 3 significant gene clusters after Bonferroni correction (*p* value < 0.05), meaning more than 3 significant sets of genes presenting the same expression profiles across the treatment conditions. The top 3 were selected for further analysis and Fig. 7a shows that the expression levels of DEGs among most clusters is gradually decreasing with time, except cluster 3 for the high concentration in colon and cluster 3 for the low concentration in SI.

The list of DEGs that clustered over time was generated and applied on NetworkAnalyst (Zhou et al. 2019) to perform an enrichment network analysis. The lists of genes obtained from the two most significant clusters, which present the same gene expression profiles, were combined, providing a more complete enrichment network (Fig. 7b). The third gene cluster obtained in STEM, and derived from both organoids, provided with less interesting networks as they were unrelated to 5-FU or intestinal biological pathways, thus they were not considered for further analyses. In the colon, at 10 µM, the most highly enriched and significant pathways were cell cycle, one carbon metabolism (pool by folate) and DNA replication. Considering 100 µM, cell cycle and biosynthesis of amino acids were the most enriched pathways, which are also linked to the effects of 5-FU on RNA synthesis. In turn, at 1000 µM, the number of enriched pathways increased significantly (Fig. 7b), being metabolic pathways the most relevant one, followed by cell cycle, DNA replication, biosynthesis of amino acids and carbon metabolism. At this concentration, pentose phosphate pathway and TCA cycle were also observed. The results for the SI were rather different from those for the colon. Firstly, number of enriched pathways in colon was higher than in SI, for all concentrations, which was expected in view of the results already discussed above. At 10 µM, the most highly enriched pathways were cell cycle and p53 signalling pathway. At 100 µM cell cycle and p53 signalling pathway still prevailed along with fork-head box transcription factors (FoxO) signalling pathway. Finally, at 1000 µM, metabolic pathways were also the most enriched, as observed in the colon, followed by glycerolipid metabolism, regulation of actin cytoskeleton and carbon metabolism (Fig. 7b). Likewise colon, TCA cycle and pentose phosphate pathway were observed as considerably affected pathways.

The most significantly affected genes derived from time-dependent clusters represented in Fig. 7 are completely different between colon and SI organoids, suggesting a tissue-specific response to 5-FU. The 5 most altered DEGs for both colon and SI are listed in Table 3. The main pathways in which these genes are involved and the diseases to which they have been associated are also described. Moreover, all the DEGs were downregulated after exposure to 5-FU as compared to the controls. In the colon, the 5 DEGs that stood out were *SLC9A3*, involved in signal transduction; *AQP6,* an aquaporin encoding gene; *JMJD4,* involved in the termination of protein biosynthesis via the eukaryotic translation termination factor 1 (ETF1); *FIBCD1,* involved in endocytosis; and *FASTK*, which has a role in FAS-mediated apoptosis. Alterations in these genes have been associated with diarrhoea; colon and gastric cancers (Table 3). On the other hand, in SI it is observed *KIF14*, involved in signalling by RHO GTPases; *AQP12B,* an aquaporin encoding gene; *SMIM10L2A,* associated with p-53 signalling pathway; *KIF26B,* which mediates cell signalling pathways; and lastly, *TMEM 187,* whose function is still uncertain. The diseases associated fall into GI cancers and celiac disease, whose classic intestinal symptoms include diarrhoea and weigh loss (Table 3).

**Table 3:** The most significantly altered DEGs selected after analysis with STEM, considering clusters 1 and 2, and the exposure to the high concentration over time are described

Gene symbol	Name	Direction of expression (ctrl vs 5-FU)	Main pathway involved	Associated diseases
Colon				
*SLC9A3*	Solute Carrier Family 9 Member A3	↓	Transport of small molecules; signal transduction	Congenital secretory sodium diarrhoea (Dimitrov et al. 2019; Janecke et al. 2016); ulcerative colitis (Fonseca-Camarillo and Yamamoto-Furusho 2012)
*AQP6*	Aquaporin 6	↓	Transport of small molecules; aquaporin-mediated transport	Diarrhoea
*JMJD4*	Jumonji domain containing protein 4	↓	Translational termination efficiency of ETF1	Colon adenocarcinoma (Ho et al. 2018)
*FIBCD1*	Fibrinogen C Domain-Containing Protein 1	↓	Endocytosis of acetylated components	Gastric cancer (Jiang et al. 2018)
*FASTK*	Fas Activated Serine/Threonine Kinase	↓	Fas-mediated apoptosis	NA
SI				
*KIF14*	Kinesin Family Member 14	↓	Signalling by Rho GTPases	Colorectal cancer (Wang et al. 2018)
*AQP12B*	Aquaporin 12B	↓	Aquaporin-mediated transport	Diarrhoea
*SMIM10L2A*	Small Integral Membrane Protein 10 Like 2A	↓	p-53 mediated cell cycle arrest	Gastric cancer (Ji et al. 2019)
*KIF26B*	Kinesin Family Member 26B	↓	Cell signalling	Colorectal adenoma (Horpaopan et al. 2015)
*TMEM187*	Transmembrane Protein 187	↓	NA	Celiac disease (Pascual et al. 2016)

The DEGs are specific either to colon or SI. The complete name of the DEGs, as well as the main pathways in which they are involved and their associated diseases are also described

*ctrl* control; *ETF1* Eukaryotic Translation Termination Factor 1; *NA* not available; *SI* small intestine

### Evaluation of metabolomic changes in culture supernatants of exposed intestinal organoids

**Fig. 8:**
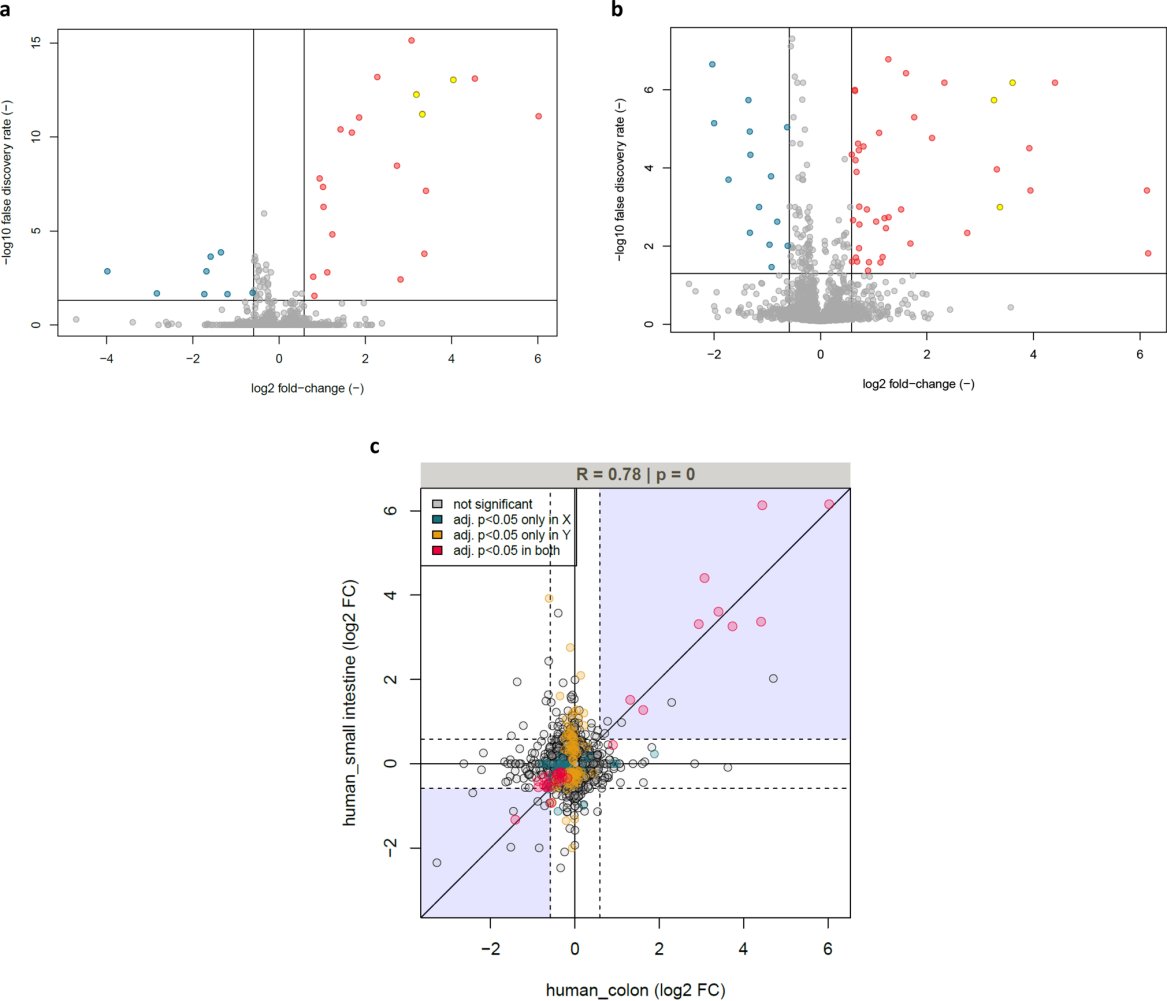

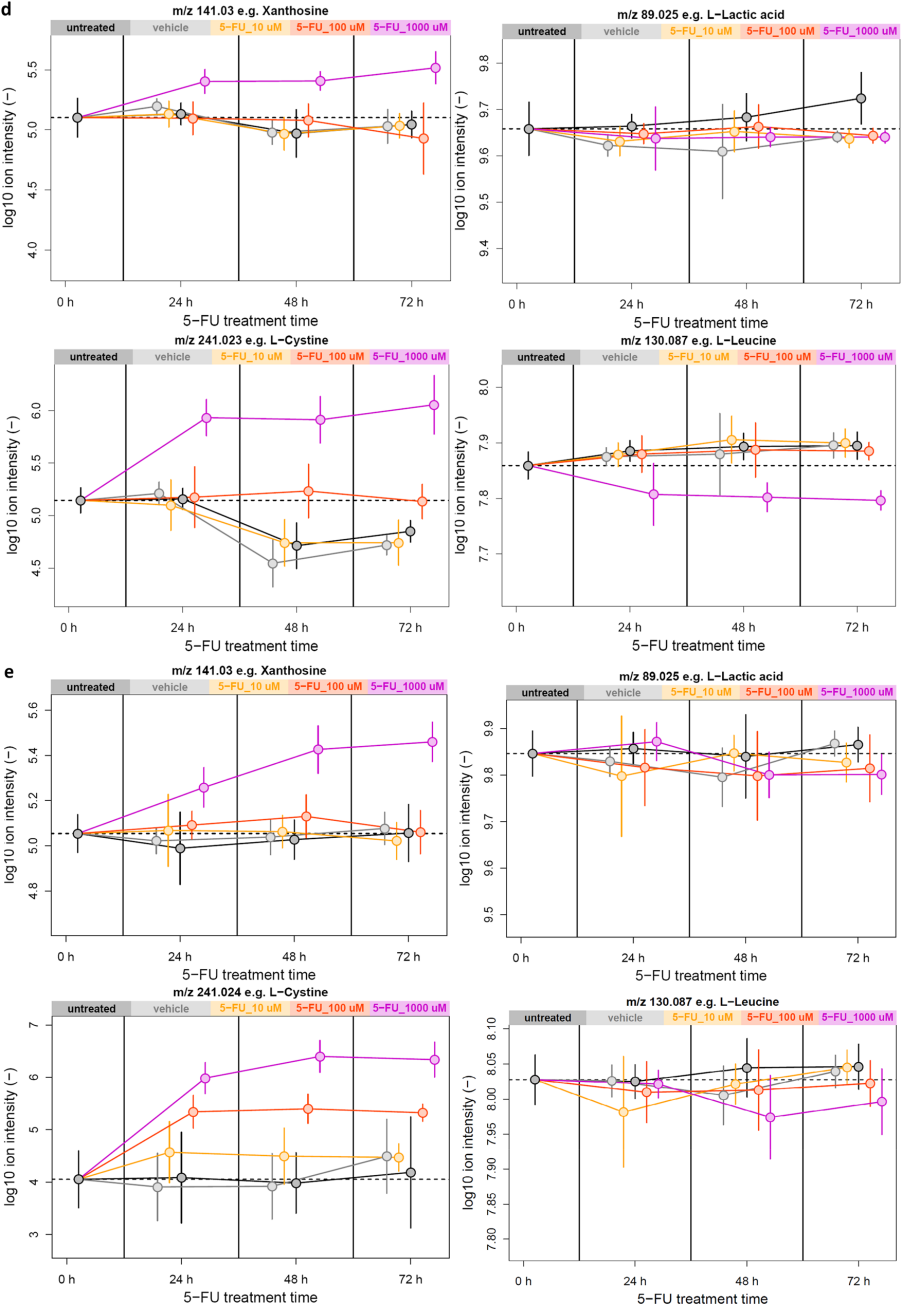
**a** Metabolic response to 1000 µM 5-FU treatment for 72 h in human colon organoid supernatants versus DMSO treatment. *p* values were calculated by unpaired *t* tests and adjusted for multiple hypothesis testing using Storey’s and Tibshirani’s method. The horizontal line indicates a false-discovery rate cut-off of 0.05, and the two vertical lines indicate a fold-change cut-off of 50%. Data points represent metabolite ions and are coloured according to fold change and false discovery rate cut-offs. Yellow ions are tentatively annotated as 5-FU itself as well as its known metabolites; **b** Same analysis as shown in panel a but for SI organoid supernatants; **c** Correlation of the log2 fold change values of metabolite ions in human colon and SI organoid supernatants as presented in panels **a** and **b**. Data points represent metabolite ions and are coloured according to the statistical significance of their response to 5-FU treatment as indicated in the legend; **d** Visualization of selected metabolite ion data from human colon organoid supernatants. Points represent the mean and error bars the standard deviation of at least 4 biological replicates. Data are horizontally off-set for improved visualization; **e** Same type of plots shown in **d**, but for human SI organoids (color figure online)

To assess the role of cellular metabolism in 5-FU-induced growth inhibition, we employed flow injection-mass spectrometry-based untargeted metabolomics (Fuhrer et al. 2011) to analyse supernatants from the same human colon and SI organoid cultures previously analysed by transcriptomics. Again, the samples comprised four different treatment groups (DMSO, 10 μM 5-FU, 100 μM 5-FU, 1000 μM 5-FU) and three time points (24 h, 48 h, 72 h) for each of the organoid models, with a minimum of four replicates per condition. In addition, control samples without any treatments were also included for each time point to assess potential DMSO effects. In colon organoids, we detected 5852 ions tentatively annotated as up to 3386 endogenous metabolites based on accurate mass. In SI organoids, 5259 ions mapping to up to 3158 metabolites were detected. Note that accurate mass-based annotations can be ambiguous as, for instance, isomers cannot be distinguished. To identify metabolite ions significantly affected by 5-FU treatment, differential analyses comparing each 5-FU treatment to its time-matched DMSO control were performed. Strongly and significantly affected metabolite ions were defined as having a fold-change of at least 50% in either direction, and a corresponding false discovery rate below 0.05. Complete statistical analysis was performed for both organoid types separately to check most relevant metabolites for each treatment condition (Supplemental Spreadsheets 1 and 2). DMSO treatment did not have a detectable effect on supernatant metabolite levels. 5-FU itself was detected as m/z 129.011 in both datasets and its levels remained stable over time for all three concentrations. As expected, in both colon and SI organoids the number of metabolite ions significantly responding to 5-FU treatment increased with increasing 5-FU treatment concentration and duration. The metabolic responses to 1000 μM 5-FU treatment are visualized in Fig. 8a and b. In general, significant metabolic responses in colon and SI organoids showed a substantial overlap, with log2 fold-changes upon 1000 µM 5-FU treatment correlating with Pearson’s *R* = 0.78 (Fig. 8c). At the 1000 μM concentration, it was observed a significant decrease in the secretion of the tricarboxylic acid cycle intermediates citric acid, malic acid and succinic acid. The secretion pattern of pyrimidine nucleosides was significantly altered as well. Other metabolic responses observed in both organoid models included changes in metabolites involved in oxidative stress management such as the disulfide cystine. Among the few model-specific responses, lactic acid was found to only deplete in SI but not in colon organoids. Conversely, levels of the essential amino acids leucine and/or isoleucine was only depleted significantly in colon organoids. Data for selected metabolite ions mentioned above are visualized in Fig. 8d and e.

### Multi-omics analysis

5-FU perturbation significantly influenced both the transcriptomic and metabolomic state of the organoids. We applied COSMOS (Casual Oriented Search of Multi-omics Space) (41) for an integrated analysis of the transcriptomics and metabolomics data to find mechanistic explanations for the observed changes.

First, the activity of the transcription factors was estimated based on a footprint method for each sample (Supplemental Figure S3). Based on the normalized enrichment score (NES), three members of the cell-cycle regulating E2F group of transcription factors (E2F1, E2F2 and E2F4) were found downregulated in all samples. Furthermore, *ZNF263*, a transcription repressor also involved in the regulation of cell growth and differentiation (Okubo et al. 1995), was strongly upregulated in all colon samples and the activation increased over time and for a higher concentration of 5-FU.

Secondly, we mapped the deregulated transcription factors and the measured metabolites to the prior knowledge network (PKN) of COSMOS, which contains 117,065 interactions among 37,863 proteins and metabolites. To make the prior knowledge specific to colon and SI organoids, the interactions that contained proteins for which the genes were not expressed in any measured conditions were removed (see details in methods 4.11). In total, 17,180 and 16,078 genes were found to be expressed at least in one condition in SI and colon samples, respectively. After removing the non-expressed nodes from the PKN, it was observed a SI-specific PKN containing 61,203 interactions among 16,972 nodes, and a colon-specific PKN with 58,954 interactions among 16,516 nodes. The measured metabolites and the previously estimated transcription factors were mapped to the PKNs, which resulted in 391 unique, significantly changing (q-value < 0.05) metabolites mapped to the PKN. Furthermore, the PKN contained 88 of the 113 strongly deregulated (abs(NES) > 1.96) transcription factors.

COSMOS was then run in the forward mode in order to explain the changes in metabolites based on the estimated changes in the transcription factor activity. This resulted in multiple networks for each sample (Supplemental Figure S4), which were aggregated. In these aggregated networks of each sample, the edges were weighted based on the number of times they appeared across the multiple solutions. This way, the edge weight served as an essentiality score, which shows how important an edge is to explain the observed activity patterns of transcription factors and metabolites.

**Fig. 9:**
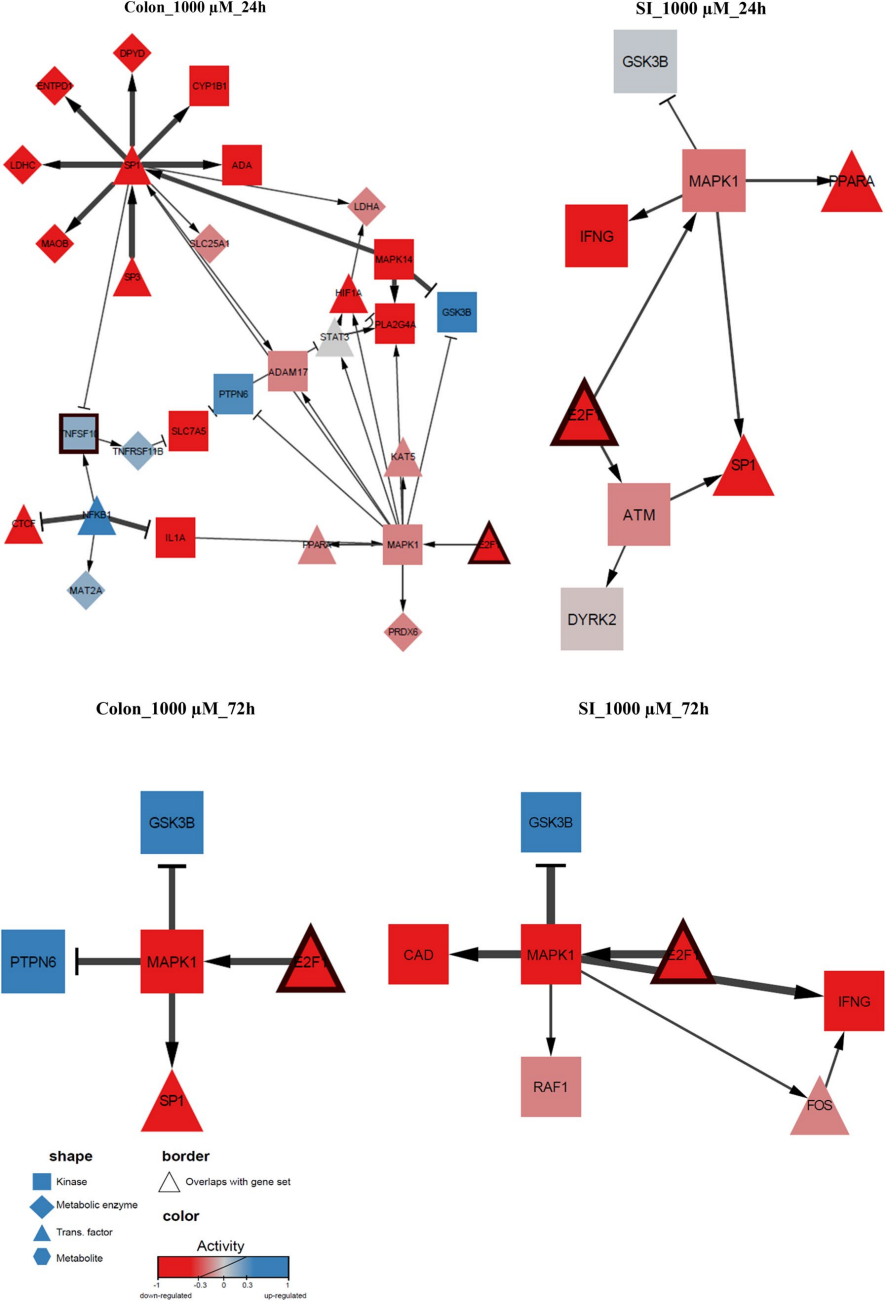
Transcription factor E2F1 as key node in COSMOS networks correlated with cell cycle regulation and apoptosis, obtained after multi-omics integration of 5-FU data and the overlap between COSMOS and gene-set, for colon and SI organoids exposed to 1000 µM 5-FU during 24 h and 72 h. → induce; − −| inhibit

The above gene set enrichment analysis showed that 5-FU influences the genes involved in cell cycle and apoptosis, among other relevant pathways (Table 2). To explore this mechanism based on the results of COSMOS, the neighbourhood of those proteins in the COSMOS network that also appeared in the gene sets were visualized. As a result, it was observed that E2F1, a strongly down-regulated transcription factor, which plays a role in cell cycle, DNA damage checkpoints and apoptosis (Dubrez 2017), appeared consistently in the networks of multiple samples. The obtained E2F1-related networks can be visualized in Fig. 9, in which only the highest concentration for both organoids was considered since the changes were more significant. In these networks, transcription factor E2F1 was found to influence MAPK1 signalling pathways as well as to be related to *TP53 and TNFSF10* genes described above.

## Discussion

One of the main goals of this study was to apply a novel 3D in vitro model and evaluate its potential use in toxicological testing and translational research. To achieve this, 5-FU toxic effects were evaluated in colon and SI organoids. The 3D in vitro model used in this study was developed to better resemble the human tissues in comparison to current standard 2D in vitro models. Cultures of organoids were derived from human healthy tissue biopsies and showed features that resemble the intestines, as reported previously (Mahe et al. 2013; Sato et al. 2011; Zachos et al. 2016). Both types of organoids were exposed to concentrations of 5-FU derived from PBPK modelling to mimic the in vivo concentrations following clinically relevant dosing.

In general, changes in expression of genes and biological pathways were quite similar in colon and SI organoids in response to 5-FU (Table 1). Despite the similarities between these two organs, the number of genes affected by 5-FU was higher in the colon than in SI organoids. Differences between the organoids were also observed in some pathways affected by 5-FU. This concerns the absence of pathways related to ATP synthesis in SI, which was concordant with the ATP-based viability measurements, as well as metabolism-related pathways, which appeared to be more strongly affected in colon than in SI organoids. Next, apoptosis was similarly affected in both organs, but the alteration in the gene expression in the caspase 3/7 assay became more statistically significant in colon as the concentration and duration of exposure increased. Cell cycle was one of the most affected pathways in both organoids, along with closely related pathways, such as DNA synthesis and repair, whose downregulation was observed in especially a concentration–dependent manner. This is concordant with the known main mechanism of action of 5-FU, namely the incorporation of fluoronucleotides into DNA and RNA molecules (Longley et al. 2003), which perturbs DNA and RNA synthesis (Lee et al. 2014; Longley et al. 2003). Consequently, this may affect cell survival and proliferation particularly when the duration and concentration of the exposure are too high to provide time for recovery and regeneration.

We compared the trend of alteration of gene responses involved in either ATP synthesis or apoptosis with viability and caspase 3/7 activation to better understand the effects of 5-FU. The transcriptomic data in Table 2 showed that ATP synthesis and the respiratory electron transport chain are only affected in the colon. Results obtained from the viability assays showed that the variation in ATP levels was much stronger in the colon than in the SI, for which the viability remains almost unchanged until the higher concentration and duration are reached, in contrast to what was observed for the colon organoids (Fig. 3). In concordance with these observations, the fold change of the DEGs was higher in the colon than SI (Figs. 5 and 6). The most perturbed genes involved in ATP synthesis were *ACAD9, UCP2, NDUFAF3, SLC25A27* and *ATP5F1D*. These genes are involved in the assembly of mitochondrial complex I proteins (*ACAD9* and *NDUFAF3*), mitochondrial uncoupling proteins (*UCP2* and *SLC25A27*) and as part of ATP synthase subunit (*ATP5F1D*). Therefore, proteins encoded by those genes are important components of mitochondrial function as they play a key role in cellular respiration, a well-known mechanism that results in the production of energy (ATP) and oxygen that keep cells alive (Alberts 2015). As duration and concentration of 5-FU exposure increased, expression of those genes decreased, indicating impairment in ATP production via mitochondrial respiratory electron transport chain, which could also be related to mitochondrial dysfunction. The decrease in the levels of ATP in the organoids was also a continuous process throughout the exposure to 5-FU, particularly in the colon organoids. This demonstrates that ATP production and mitochondrial function in the colon organoids were more strongly affected by 5-FU toxicity than in the SI organoids.

Furthermore, the results showed that apoptosis was activated as a concentration duration–response effect in both organoids. Apoptosis seemed to be more relevant in colon organoids at 10 µM but at the 1000 µM, it became more activated in the SI. Additionally, the caspase 3/7 assay clearly showed that concentration-effect is more significant than time-effect in colon organoids. On the other hand, in caspase 3/7 activation in SI organoids, the time-effect was more prominent than the concentration-effect, thus remaining almost unchanged until the high concentration was reached (Fig. 3). Furthermore, pro-apoptotic genes were mostly upregulated in both organoids, particularly the genes, *CASP3/6/7/8/10, FAS, and TNFSF10, TRAIL and BAX*. These genes are involved in signalling pathways that induce apoptosis, p53 and NF-kB. Even though both intrinsic and extrinsic seem to have been activated, the latter is prevailing via the FAS-ligand mechanism and subsequent activation of caspases that lead to the also observed DNA degradation, nuclear breakdown and cell shrinkage (Shalini et al. 2015). In support of these results, downregulation of anti-apoptotic genes was found, with the only exception of *BIRC3,* which could represent the fraction of cells still fighting against 5-FU effects*.* Despite up-regulation of *BIRC3*, the overall cell survival mechanisms were not as strong, hence activation of apoptosis prevailed. The gene plots represented in Fig. 6 show that the perturbation in the expression of those genes was higher in the colon organoids. These results support the hypothesis of a tissue-specific response when colon and SI cells are exposed to the same drug at the same concentration.

Pathways related to p53 signalling seemed to play an important role in both organoids as well. In colon organoids, p53 signalling pathways were strongly affected in the initial time of exposure at the low concentration, whereas in SI organoids, this pathway became more significantly affected at later time points and higher concentrations. Indeed, it has been reported that p53, as well as NF-kB, are key factors in mediating gene responses, particularly those involved in cell cycle arrest, apoptosis, ATP production and recruitment of inflammatory components including cytokines, which in turn, can be related to symptoms of toxicity and inflammation (Chang et al. 2012; Pritchard et al. 1998).

Performing further time-dependency analysis with the STEM tool confirmed that cell cycle, DNA replication, p53 signalling and metabolism were affected in both colon and SI. Additionally, new pathways were identified as being affected after the STEM analysis. In the colon organoids, effects were found associated with one carbon metabolism mediated by folate and biosynthesis of amino acids. In the SI, FoxO signalling pathway was affected, which is responsible for regulating gene expression; glycerolipid metabolism and regulation of actin cytoskeleton, involved in tissue damage/repair (Lee and Dominguez 2010). Tissue specific DEGs derived from the most significant time-dependent clusters (Fig. 7a and b) were investigated at the high concentration, and, as a result, colon and SI presented a distinct set of 5 most affected genes (Table 2). The 5 DEGs found significantly altered in colon organoids include *SLC9A3, AQP6, JMJD4, FIBCD1* and *FASTK*. *SLC9A3* is a solute carrier encoding gene involved in signal transduction and as a result of the exposure to 5-FU, it was found downregulated over time and concentration. Indeed, *SLC9A3* has been found downregulated in patients with ulcerative colitis (colon inflammation) (Fonseca-Camarillo and Yamamoto-Furusho 2012) and its loss of function has been linked to diarrhoea disorders (Dimitrov et al. 2019; Janecke et al. 2016). Since 5-FU is known to cause intestinal inflammation and diarrhoea, this gene may be an indicative of the risk of colon toxicity. *AQP6* is an aquaporin encoding gene mainly involved in water transfer across membranes. Its downregulation due to 5-FU exposure, along with *SLC9A3*, can contribute to the development of diarrhoea. In turn, overexpression of *JMJD4* and *FIBCD1* has been linked to cancers of the GI tract (Ho et al. 2018; Jiang et al. 2018). Furthermore, *FIBCD1* might play a role in inflammation and immune responses as well (von Huth et al. 2018). In this study, downregulation of these two genes was observed after exposure of organoids derived from healthy human cells to 5-FU, which can be regarded as a specific response of healthy cells towards the drug. Regarding *FASTK*, it encodes a member of the serine/threonine protein kinase family and regulates *FAS* alternative splicing that gives rise to isoforms that do not promote apoptosis (Lin et al. 2016). Downregulation of *FASTK* is thus consistent with the increasing activation of apoptosis in exposed cells. Regarding SI organoids, the most affected genes were also downregulated due to 5-FU, namely *KIF14, KIF26B, AQP12B, SMIM10L2A* and *TMEM187* (Table 3). *KIF14* and *KIF26B* belong to the kinesin family encoding genes and are involved in cell signalling. In particular, KIF14 is known to participate in the Rho GTPases signalling pathways, which are important in several cellular functions such as cell cycle, cell dynamics and organelle development (Bustelo et al. 2007). Overexpression of both genes has been linked to the development and poor prognosis of colorectal cancer (Horpaopan et al. 2015; Wang et al. 2018). Likewise, Rho GTPases such as RhoA, RhoC and Rac1 were found to be upregulated in colorectal carcinoma (Haga and Ridley 2016; Leve and Morgado-Diaz 2012). In contrast, in healthy SI cells exposed to 5-FU, expression levels of those genes decreased. In the SI also a different aquaporin encoding gene was found, *AQP12B,* although it has the same cellular function. Another interesting gene was *SMIM10L2A*, also known as a long noncoding RNA *LINC00086*, whose function has been associated with regulation of gene expression via p53 signalling pathway (Leveille et al. 2015). Overall, this pathway was indeed more affected in SI than in colon at higher concentrations. Lastly, *TMEM187* encodes a transmembrane protein whose biological function remains unclear. Apart from celiac disease, a chronic immune disease of the small bowel (Pascual et al. 2016), there is no other link between this gene and SI or 5-FU exposure. Nevertheless, *TMEM187* may still be valuable in evaluating 5-FU toxicity in SI.

The metabolomic analyses of the supernatant of exposed intestinal organoids showed that colon and SI presented very similar metabolic responses. As expected, the effect of 5-FU became more significant as the concentration and duration increased. In both colon and SI supernatants, considering exposure to 1000 µM 5-FU, the decrease in the levels of secreted TCA cycle intermediates can potentially be reflective of impaired cell growth, which is consistent with the data described above. It was also observed significant alterations in the secretion of pyrimidine nucleosides, which is in line with previous results as well as earlier reports of 5-FU inducing nucleotide overflow in human cancer cell lines (Ser et al. 2016). Responses to oxidative stress were also significant and consistent with general cellular stress exerted by 5-FU. Moreover, and likewise the transcriptomic data, colon and SI organoids presented tissue-specific metabolic responses. In colon organoids, a significant depletion of leucine and isoleucine was observed, supporting previously reported links in hepatocarcinoma cell lines between branched-chain amino acid metabolism and 5-FU efficacy (Nishitani et al. 2013). In turn, only SI presented a significant depletion of lactic acid indicating that 5-FU might cause a stronger inhibition of the glycolytic pathway in this tissue.

Lastly, multi-omics integration of DEGs and metabolites was performed. As a result, relevant and complex networks were obtained, of which the ones that involve transcription factor E2F1 stood out as most consistent across the study. At the highest concentration and latest time point, E2F1 and downstream proteins and metabolites were generally more significantly downregulated, particularly MAPK1 kinase, involved in several cellular processes including proliferation and differentiation (Yoon and Seger 2006). Also up-regulation was observed as in the case of GSK3B, a negative regulator of glucose homeostasis and involved in inflammation, mitochondria dysfunction and apoptosis (Patel and Woodgett 2017). Transcription factor E2F1 plays an essential role in regulation of cell cycle and apoptosis (Shats et al. 2017), as well as in DNA damage response checkpoints (Stevens and La Thangue 2004). Therefore, and dependent on the stimuli, E2F1 can either promote transcription of cell cycle genes, favouring cell growth and proliferation, or of cell death genes, leading to apoptosis (Dubrez 2017; Shats et al. 2017). In this study, 5-FU caused the downregulation of transcription factor E2F1, which led to impairment of cell cycle progression, which is in line with the observed downregulation of cell cycle DEGS. In turn, 5-FU effects on this transcription factor favoured upregulation of apoptotic genes including *TP53* and *TNFSF10* (Figs. 5, 6 and 9), consequently activating p53 signalling pathways and apoptosis, as described above.

Taken all together, the results obtained from phenotypic, transcriptomic and metabolomic data demonstrate that colon organoids are more sensitive to 5-FU as their responses were stronger than those of SI organoids. For this reason, we hypothesize that the colon is more affected by the toxic effects of the drug. Differences between colon and SI also support the hypothesis of tissue-specific responses that could be useful in future screening studies. Novel mechanisms prevailing in colon are the downregulation of biosynthesis and transport of small molecules such as amino acids, specifically leucine and isoleucine, and ETF1 efficiency in mRNA translation, which can negatively impact protein production. On the other hand, in SI, new mechanisms were related to Rho GTPases and FoxO cell signalling pathways, regulators of important cellular functions including glucose metabolism, which can be linked to alterations in lactic acid levels in the supernatant, and resistance to oxidative stress (Bustelo et al. 2007; Lee and Dominguez 2010). Additionally, one of mechanisms by which 5-FU induces intestinal toxicity is likely to involve transcription factor E2F1 activity. Therefore, this study reveals a new set of molecular mechanisms and gene profiles that have not yet been associated with 5-FU.

Only a few studies have established 5-FU mechanisms of toxicity at the level of transcriptomic responses in humans (Rodrigues et al. 2019). Two of the most significantly altered DEGs found in SI organoids were previously reported, namely *TYMS* (Braun et al. 2009) and *EML2* (Del Rio et al. 2007). The first one codes for an important enzyme in DNA synthesis, thymidylate synthase, which generates thymidine. Deregulation of *TYMS* leads to an imbalance of deoxynucleotides, and consequently, it causes DNA damage, a well-known 5-FU mechanism of action. *EML2* is involved in cell signalling that influences cell growth. Nevertheless, those two studies with human interventions included only tumour colon samples and patients also received other drugs in addition to 5-FU (Braun et al. 2009; Del Rio et al. 2007). Therefore, validation of our findings with human clinical data is still rather limited.

In conclusion, this study demonstrates that the organoid-based 3D in vitro approaches, informed by modelling and simulation, are able to provide new insights in potential 5-FU mechanisms of toxicity in intestines, in addition to what is already known. This in vitro model may potentially contribute to the improvement of quantitative in silico models that aim to predict adverse effects in the intestines of new compounds under development for future treatment of cancer patients. Future studies should include the evaluation of functional endpoints and transcriptomic responses in cancer patients taking 5-FU monotherapy. This is crucial to demonstrate to what extent intestinal organoids more accurately reflect the responses in patients than other cell models. Ultimately, this new in vitro model may be an alternative to perform drug experimental studies so that the use of animal studies can be minimized.

## Supplementary Information

Below is the link to the electronic supplementary material.

Supplementary file1 (PDF 917 KB)

Supplementary file2 (XLSX 2773 KB)

Supplementary file3 (XLSX 4022 KB)
