# Supplementary material for: New insights into the mechanisms underlying 5-fluorouracil-induced intestinal toxicity based on transcriptomic and metabolomic responses in human intestinal organoids
Source: Arch Toxicol. 2021 Jun 20;95(8):2691–718. doi: 10.1007/s00204-021-03092-2 (PMC8298376; doi:10.1007/s00204-021-03092-2)
Supplement: Supplementary file 1 — Supplementary file1 (PDF 917 KB) [file 204_2021_3092_MOESM1_ESM.pdf]

New insights into the mechanisms underlying 5-fluorouracil-induced intestinal toxicity based on transcriptomic and metabolomic responses in human intestinal organoids

Daniela Rodrigues[1*], Terezinha de Souza[1], Luke Coyle[2], Matteo Di Piazza[2], Bram Herpers[3], Sofia Ferreira[4], Mian Zhang[4], Johanna Vappiani[5], Daniel C. Sévin[5], Attila Gabor[6], Anthony Lynch[7], Seung-Wook Chung[2], Julio Saez-Rodriguez[7,8,9], Danyel G.J. Jennen[1], Jos C.S. Kleinjans[1], Theo M. de Kok[1]

[1]Department of Toxicogenomics, GROW School for Oncology and Developmental Biology, Maastricht University, Maastricht, the Netherlands.

[2]Departmnet of Nonclinical Drug Safety, Boehringer Ingelheim Pharmaceuticals Inc., Ridgefield, Connecticut, USA. MDP is currently an employee of F. Hoffmann-La Roche AG.

[3]OcellO B.V., BioPartner Center, Leiden, the Netherlands

[4]Certara UK Limited, Simcyp Division, Sheffield S1 2BJ, UK

[5]GSK Functional Genomics/Cellzome, 69117 Heidelberg, Germany

[6]Faculty of Medicine, Heidelberg University Hospital, Institute for Computational Biomedicine, Heidelberg, Germany

[7]GSK Non-Clinical Safety, Ware, SG12 0DP UK

[8]Faculty of Medicine, Joint Research Centre for Computational Biomedicine (JRC-COMBINE), RWTH Aachen University, Aachen, Germany

[9]Molecular Medicine Partnership Unit, European Molecular Biology Laboratory, Heidelberg University, Heidelberg, Germany

*corresponding author: Daniela Rodrigues, d.rodrigues@maastrichtuniversity.nl, ORCID ID: 0000-0001-5527-6627

Journal: *Archives of Toxicology*

Supplementary Information

Supplementary Table 1. Input parameters for 5-flurouracil (5-FU) human physiologically-based pharmacokinetic (PBPK) modelling in Simcyp and *in vitro* distribution modelling in SIVA (module 3). The virtual *in vitro* intracellular distribution (VIVD) model was developed to describe *in vitro* distribution in 2D cell culture systems (Fisher et al. 2019). Therefore, in this work, the model was used as an initial guide to select nominal concentrations for *in vitro* organoid studies.

**Input parameters for 5-FU kinetic modelling**		
**Physicochemical**	Molecular weight (Kim et al. 2019)	130.08 g/mol
	Lipophilicity (Kim et al. 2019)<br>(Log $P_{o:w}$)	-0.89
	Compound type (Kim et al. 2019)<br>pKa	Monoprotic acid<br>8.02
	Henry's law constant (25 °C)*	1.68×10^{-5} Pa m^3/mol
**Blood Binding**	Blood to plasma ratio (Schaaf et al. 1987)	1.09
	Fraction unbound in plasma (Celio et al. 1983; Garrett et al. 1977)	0.9
**Absorption**	M-ADAM and MechPeff model (Pade et al. 2017)<br><br>*Passive permeability ($P_{trans, 0}$) predicted from Log $P_{o:w}$*	10.3×10^{6} cm/s
**Distribution**	Rodgers & Rowland model (Rodgers et al. 2005; Rodgers and Rowland 2006)<br><br>*K_p scalar adjusted to replicate volume of distribution observed*[6]	0.2 L/kg
**Elimination**	Liver cytosol metabolism (V_{max} and K_m) (Niwa et al. 2005)	493 pmol/min/mg protein<br>3.9 µM
	Renal clearance (Heggie et al. 1987)	8 L/hr
	Additional systemic clearance<br><br>*Optimised to recover observed intravenous clearance* (Heggie et al. 1987)	37 L/hr

* Estimated from EPI Suite v4.11 using HENRYWIN v3.20 bond method

Supplementary Table 2. Human gut cell composition and culture conditions considered for 5-FU *in vitro* distribution modelling in SIVA.

**VIVD input parameters**		
**Cell composition[1]**	Fraction of cell volume comprising intracellular water	0.451
	Fraction of cell volume comprising neutral lipids	0.0487
	Fraction of cell volume comprising neutral phospholipids	0.0163
	Fraction of cell volume comprising lysosomes	0.01
	Fraction of cell volume comprising mitochondria	0.1
	pH of intracellular water	7
	pH inside lysosomes	4
	pH inside mitochondria	8
	Cell membrane potential	-41 mV
	Lysosome membrane potential	10 mV
	Mitochondria membrane potential	120 mV
	Intracellular concentration of acidic phospholipids	2.84 mg/g
**Culture conditions**	Fraction unbound in the culture medium[2]	Assumed to be 1
	pH of culture medium	7.4
	Volume of culture medium per well	100 µL
	Well diameter	6.4 mm
	Well volume	360 µL
	Culture temperature	37 °C
	Number of cells per well	3000
	Cell diameter[3]	5.3 µm

1- Human gut tissue cell composition in the Simcyp Simulator (v18r2); 2 - No albumin or lipids present in the medium; 3 - Calculated based on average organoid area (4000 µm^2) and number of cells per organoid (180 cells).

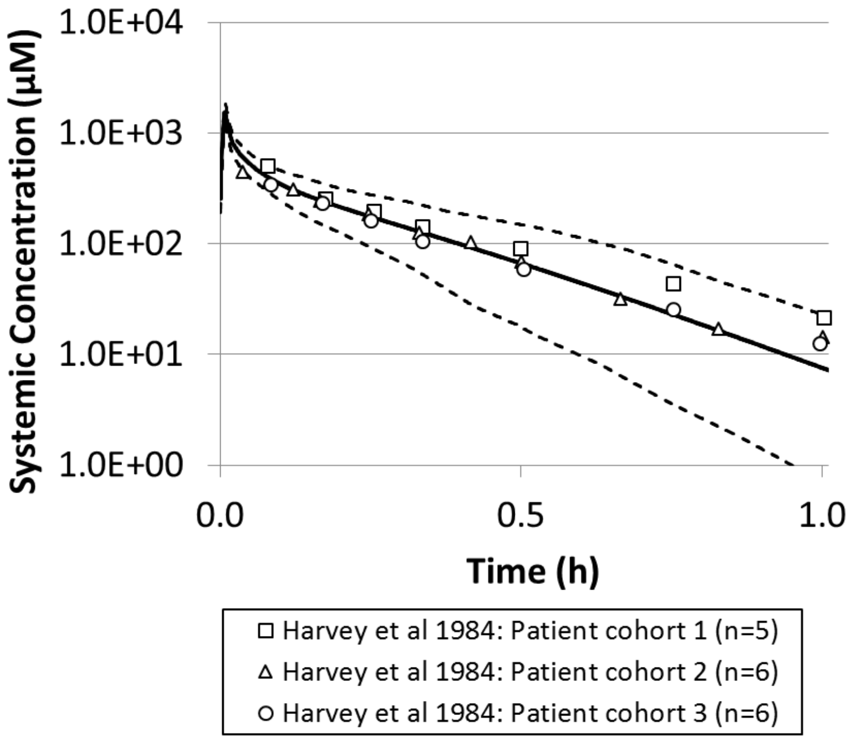

Supplementary Figure 1. Verification of the 5-FU human PBPK model: predicted (black solid line) and observed (data points) systemic plasma concentration following a 15 mg/kg (609 mg/m^2) IV bolus. The observed data was not used in the development or optimisation of the model, only in the verification of its performance. The markers show the mean concentration obtained in different studies with cancer patients (Harvey et al. 1984). The dashed lines refer to the 5th and 95th percentiles of the virtual population simulated (10 trials with 10 cancer patients each, 50% female).

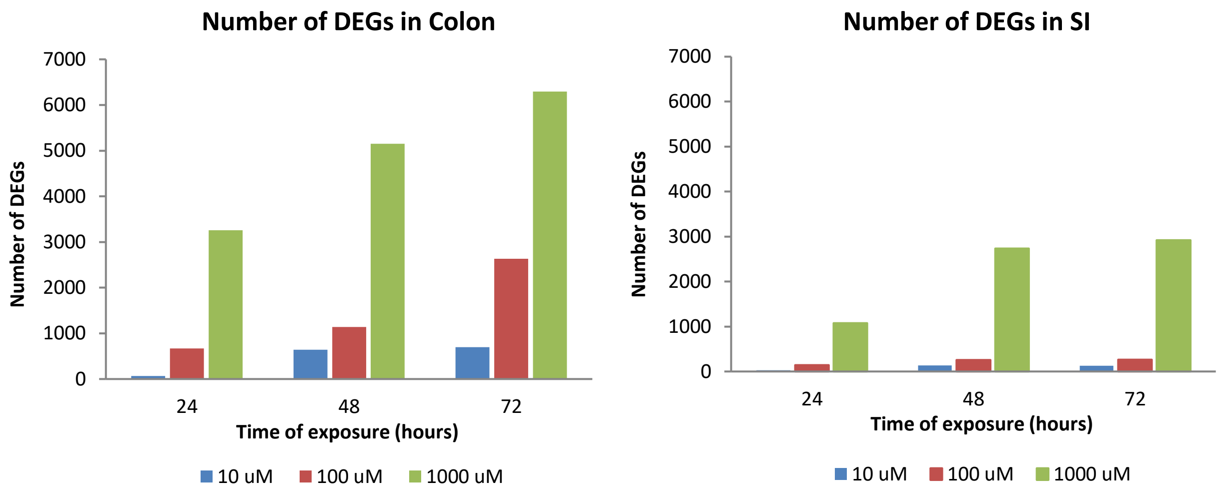

Supplementary Figure 2. Number of DEGs detected at each time point and concentration of 5-FU for colon and SI: blue corresponds to 10 µM, red corresponds to 100 µM and green corresponds to 1000 µM.. Number of DEGs was corrected using the Bonferroni method.

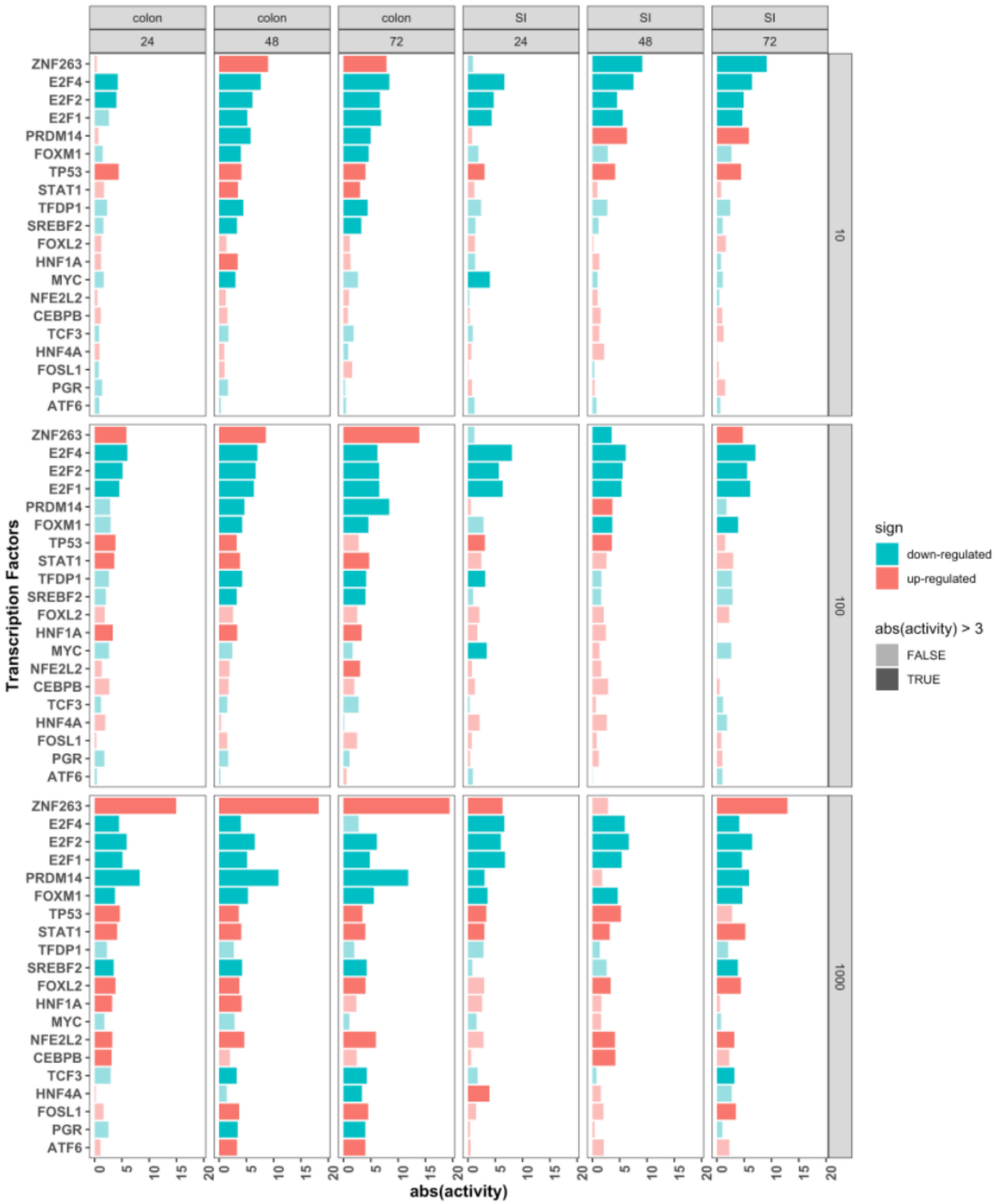

Supplementary Figure 3. Estimated transcription factor activity of colon and SI samples 24, 48 and 72 hours after treatment with 10, 100 and 1000uM of 5-FU. Upregulated TFs are shown in red and downregulated TFs are shown in blue. Strongly up- or downregulated TFs (abs(NES) >3) are shown without transparency.

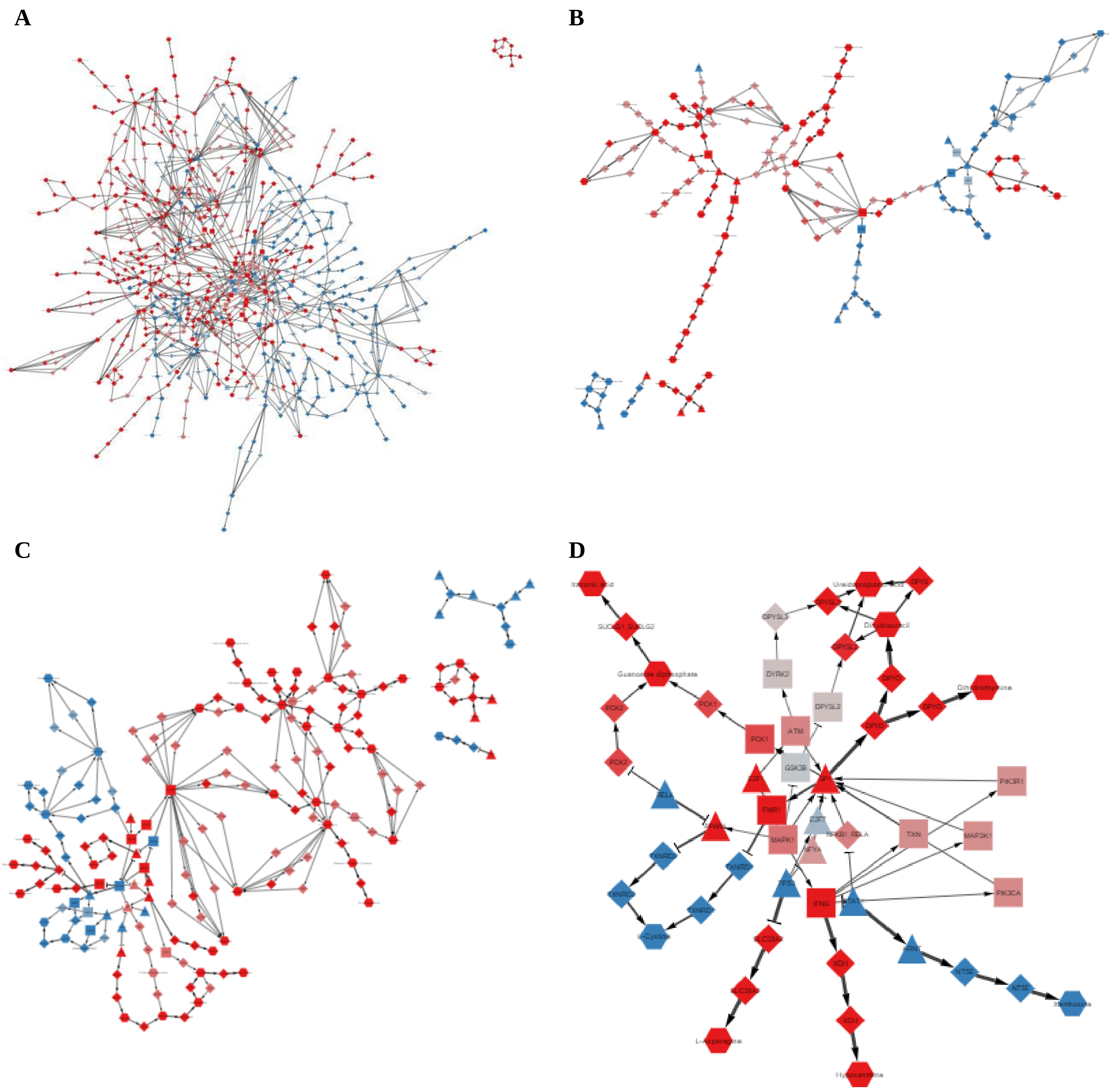
A
B
C
D
Guanosine diphosphate
DYRK2
DPYSL2
PCK1
ATM
GSK3B
PIK3R1
FMR1
MAPK1
TXN
MAP3K1
PIK3CA
IFNG
TP53
XDH
L-Asparagine
Hypoxanthine
L-Cystine
Xanthosine
DPYD
Dihydrouracil
NFYA

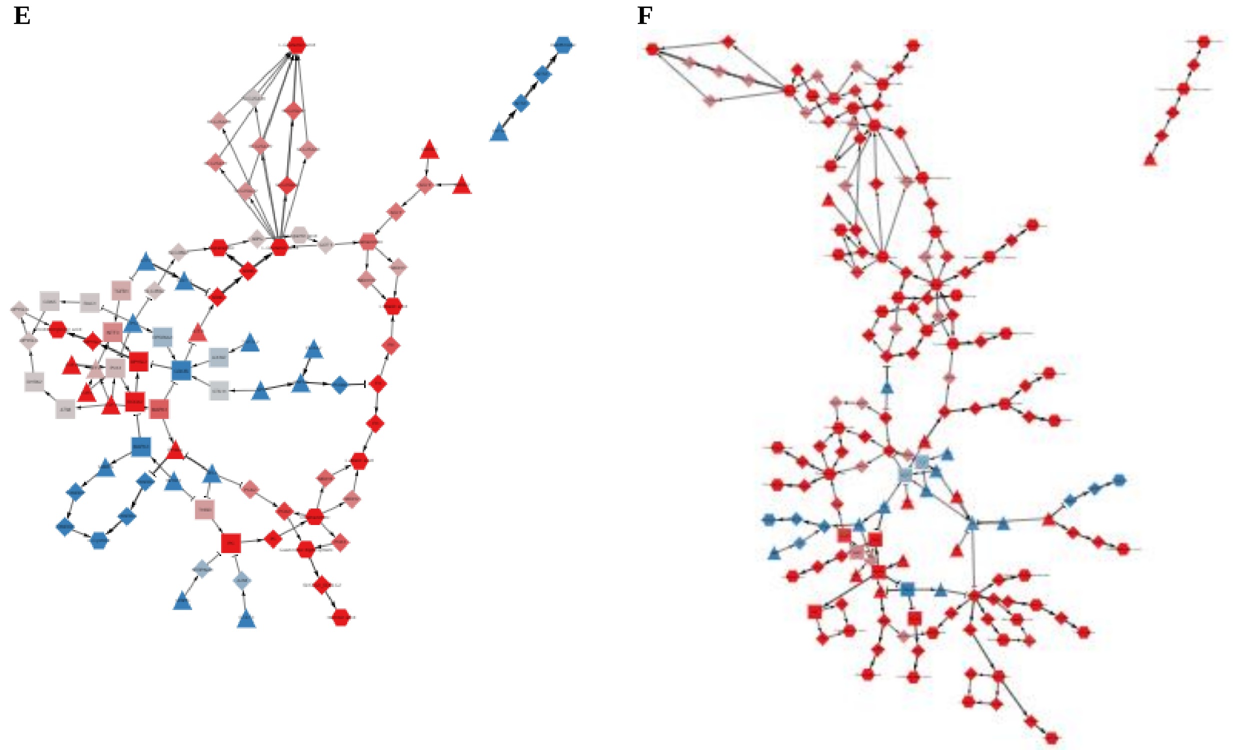

Supplementary Figure 4. COSMOS networks obtained after multi-omics integration considering the high concentration of 5-FU and all time points: A, B and C are respective to colon organoids at 24h, 48h and 72h, respectively; D, E and F are respective to SI organoids at 24h, 48h and 72h, respectively. These networks can be further explored elsewhere.
